# Astragaloside IV protects against podocyte injury via SERCA2-dependent ER stress reduction and AMPKα-regulated autophagy induction in streptozotocin-induced diabetic nephropathy

**DOI:** 10.1038/s41598-017-07061-7

**Published:** 2017-07-31

**Authors:** Hengjiang Guo, Yi Wang, Xuemei Zhang, Yingjun Zang, Yang Zhang, Li Wang, Hao Wang, Yunman Wang, Aili Cao, Wen Peng

**Affiliations:** 1 Laboratory of Renal Disease, Putuo Hospital, Shanghai University of Traditional Chinese Medicine, Shanghai, 200062 China; 2 Department of Nephrology, Putuo Hospital, Shanghai University of Traditional Chinese Medicine, Shanghai, 200062 China; 3 Department of Pharmacology, School of Pharmacy, Fudan University, Shanghai, 201203 China

## Abstract

Aberrant endoplasmic reticulum (ER) stress and autophagy are associated with diabetic nephropathy. Here we investigated the effect of astragaloside IV (AS-IV) on the progression of diabetic nephropathy (DN) and the underlying mechanism involving ER stress and autophagy in streptozotocin (STZ)-induced diabetic mice and high glucose (HG)-incubated podocytes. The diabetic mice developed progressive albuminuria and glomerulosclerosis within 8 weeks, which were significantly ameliorated by AS-IV treatment in a dose-dependent manner. Moreover, diabetes or HG-induced podocyte apoptosis was markedly attenuated by AS-IV, paralleled by a marked remission in ER stress and a remarkable restoration in impaired autophagy, which were associated with a significant improvement in the expression of sarcoendoplasmic reticulum Ca^2+^ ATPase 2b (SERCA2b) and AMP-activated protein kinase α (AMPKα) phosphorylation, respectively. Knockdown of SERCA2 in podocytes induced ER stress and largely abolished the protective effect of AS-IV, but had no obvious effect on the expression of autophagy-associated proteins. On the other hand, blockade of either autophagy induction or AMPKα activation could also significantly mitigate AS-IV-induced beneficial effect. Collectively, these results suggest that AS-IV prevented the progression of DN, which is mediated at least in part by SERCA2-dependent ER stress attenuation and AMPKα-promoted autophagy induction.

## Introduction

Diabetic nephropathy (DN) is a diabetes-induced microvascular complication that leads to end-stage renal disease (ESRD)^1^, with the progressive albuminuria as the hallmark due to compromised glomerular filtration barrier (GFB)^2^. Podocytes, highly differentiated glomerular epithelial cells, are particularly imperative for maintaining GFB function and alterations in podocytes are associated with DN development^3^. Despite the crucial importance of podocytes, the mechanism underlying podocyte injury has not been well clarified. Thus, it is important to better understand the pathogenesis of podocyte injury in order to develop new therapeutic targets for DN.

Endoplasmic reticulum (ER) stress is evoked by the accumulation of aberrant proteins in the cells due to ER dysfunction under pathological conditions, followed by the activation of an adaptive response-unfolded protein response (UPR)^4, 5^. UPR is regulated by three ER-resident transducers: activating transcription factor (ATF) 6, protein kinase R-like ER kinase (PERK), and inositol-requiring enzyme 1 (IRE1)^5, 6^, which can also induce cell apoptosis through the mediations of their downstream targets such as CCAAT/enhancer-binding protein (C/EBP) homologous protein (CHOP), c-Jun N-terminal kinases (JNK) and caspase 12 when ER stress is prolonged or excessive^5, 6^. Accumulating body of evidence implicates that ER stress is a major contributor to many kinds of kidney diseases, including DN^4^. Elevated ER stress induced podocyte apoptosis while ER stress inhibition attenuated podocyte apoptosis *in vivo* and *in vitro* ^7, 8^. The sarco/endoplasmic reticulum Ca^2+^-ATPase (SERCA), which maintains a steep Ca^2+^ concentration gradient between the cytosol and ER lumen^9^, has emerged as a key regulator of ER stress and their activities or expression was found compromised in islets^10, 11^, heart^12, 13^ and liver^14, 15^ under diabetic conditions.

On the other hand, autophagy is another adaptive response under various stress conditions in which cellular protein aggregates and damaged organelles are degraded via the lysosomal pathway to maintain intracellular homeostasis^16, 17^. Autophagy plays an important role in human health and diseases, including kidney injury^18, 19^. Growing evidence reveals that impairment of autophagy in the kidney is involved in the pathogenesis of DN and autophagy restoration may be renoprotective^20, 21^. Importantly, podocytes exhibit high levels of basal autophagy^22, 23^, indicating autophagy serves as an essential maintainer of podocyte cellular homeostasis. Autophagy deficiency in podocytes exacerbates proteinuria in DN^24^. These studies highlight the significance of constitutive and induced autophagy in protecting against podocyte injury and proteinuria. The mammalian target of rapamycin (mTOR) signaling has been reported to negatively regulate autophagy induction^16, 25^, while AMP-activated protein kinase (AMPK) activation may inhibit mTOR signaling pathway and promote autophagy in many kinds of cells^26^.

Astragaloside IV (AS-IV), a saponin purified from Astragalus membranaceus (Fisch) Bge, has exhibited various pharmacological activities in protecting renal function, such as anti-inflammation^27^, anti-oxidative stress^28^, inhibiting renal tubulointerstitial fibrosis^29^ and anti-ER stress^30^. Though AS-IV has been reported to protect podocytes and prevent DN development by inhibiting ER stress in type 1 diabetes, the underlying mechanism for AS-IV-reduced ER stress warrants further investigation. In addition, whether autophagy induction is involved in the renoprotective function of AS-IV remains unknown. Thus, this work aims to explore the mechanism by which AS-IV attenuates ER stress and the role of autophagy in the action of AS-IV in STZ-induced type 1 diabetic mice *in vivo* and in high glucose (HG)-cultured podocytes *in vitro*.

## Results

### AS-IV prevented the progression of DN in STZ-induced diabetic mice

**Figure 1:**
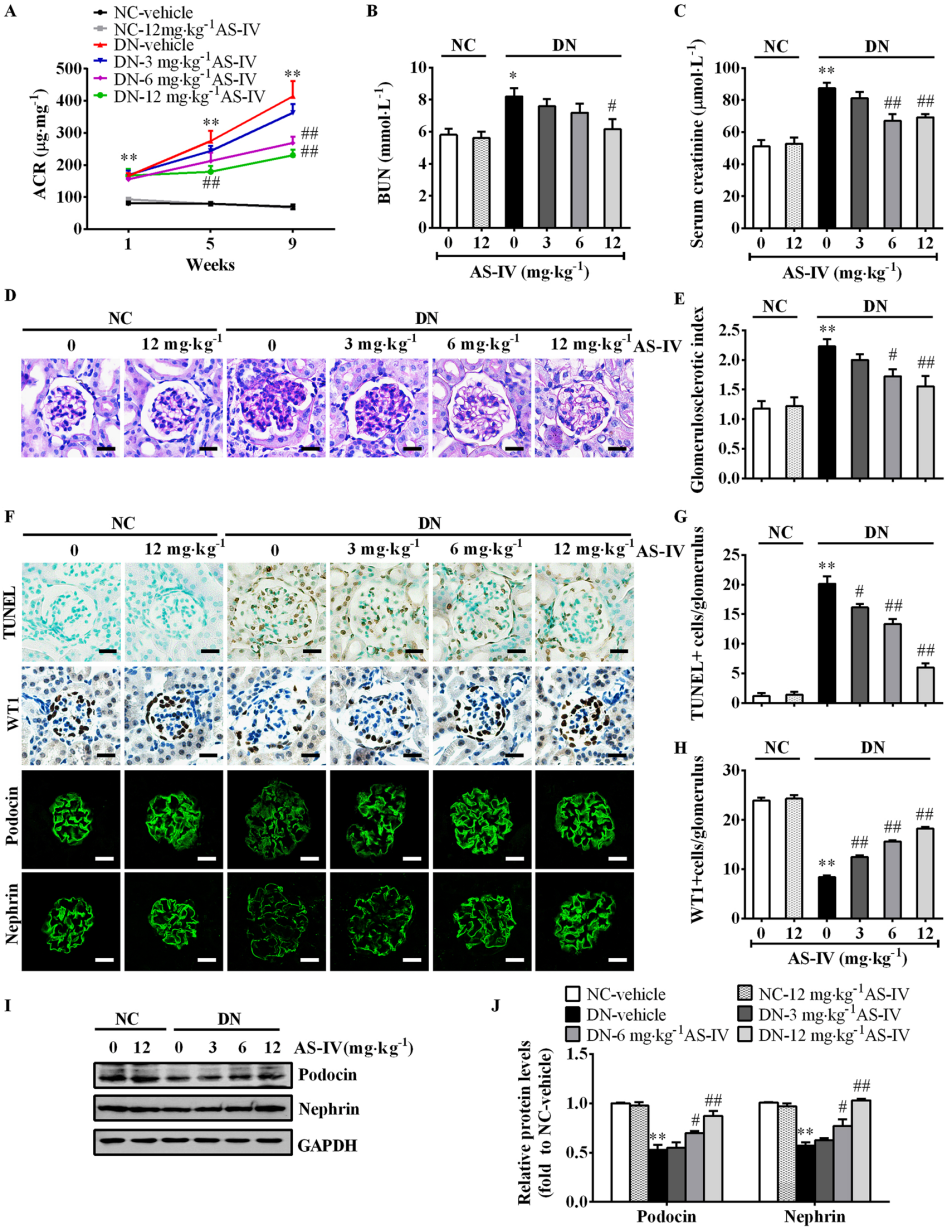
AS-IV prevented the progression of DN in STZ-induced diabetic mice. 8-week-old C57BL/6J mice were injected intraperitoneally with 100 mg·kg^−1^ STZ for 2 consecutive days in the first week (week 0). 1 week after STZ injection (week 1), the mice were treated with AS-IV at the indicated doses for 8 consecutive weeks. Urinary albumin to creatinine ratio (ACR) was measured at 4-week intervals (**A**). Serum BUN (**B**) and serum creatinine (**C**) were detected at the end of the study. (**D**) PAS staining. Scale bars, 10 μm. (**E**) Glomerulosclerotic index based on PAS staining. (**F**) Representative images of TUNEL staining, immunohistochemistry staining for WT-1 and immunofluorescence staining for Podocin and Nephrin. Scale bars, 10 μm. (**G**) Quantification of TUNEL-positive cells in each group. Data was expressed as TUNEL-positive cell number per glomerulus. (**H**) Quantification of podocyte number in each group. Results were represented as WT-1-positive podocyte number per glomerulus. (**I**,**J**) Representative immunoblots (**I**) and densitometric quantification (**J**) of Podocin and Nephrin expression in total lysates of kidney cortex from each group. The original blots for panel I were presented in Supplementary Figure S2. Data are expressed as mean ± SEM. n = 10–12 for A–C and n = 4–6 for D–J. ^**^ *P* < 0.01 compared to NC-vehicle group; ^*#*^ *P* < 0.05, ^*##*^ *P* < 0.01 compared to DN-vehicle group. One-way ANOVA and Newman-Keuls multiple comparisons test (**A**–**C**,**E**,**G**,**H**,**J**). NC, non-diabetic control. DN, diabetic nephropathy.

C57BL/6J mice were rendered diabetic by using a two-dose regimen of STZ as reported^31^. The STZ-injected mice developed hyperglycemia (blood glucose > 350 mg·dL^−1^) 1 week after STZ treatment. At this point the mice were randomly allocated into 4 groups and treated respectively with vehicle, or 3, 6, and 12 mg^−1^·kg^−1^ day^−1^ AS-IV for 8 weeks, with nondiabetic mice receiving vehicle or 12 mg^−1^·kg^−1^ day^−1^ AS-IV as controls. The hyperglycemia persisted during the 8-week period and all STZ-induced diabetic mice failed to gain weight during the 8 weeks (Table 1). As expected, DN-vehicle mice developed robust progressive albuminuria (expressed as urinary albumin to creatinine ratio (ACR)) and AS-IV prevented the development of diabetes-induced albuminuria in a dose-dependent manner (Fig. 1A) without altering the blood glucose levels and body weight (Table 1). Consistently, both blood urea nitrogen (BUN) and serum creatinine levels were markedly elevated in DN-vehicle group relative to NC-vehicle group, which were significantly reduced by AS-IV treatment in a dose-dependent manner (Fig. 1B,C).

**Table 1:** Fasting blood glucose and body weight during the treatment period.

Groups	Blood glucose (mg·dL^−1^)			Body weight (g)		
	Week 1	Week 5	Week 9	Week 1	Week 5	Week 9
NC-vehicle	108.3 ± 7.7	102.3 ± 5.6	138.1 ± 7.1	19.6 ± 0.37	20.7 ± 0.21	24.3 ± 0.61
NC-12 mg·kg^−1^ AS-IV	102.3 ± 5.6	108.3 ± 7.7	133.6 ± 5.2	19.3 ± 0.26	20.4 ± 0.16	23.7 ± 0.53
DN-vehicle	438.3 ± 16.2^**^	532.7 ± 26.0^**^	503.0 ± 27.5^**^	18.4 ± 0.45	17.2 ± 0.20^**^	17.1 ± 0.28^**^
DN-3 mg·kg^−1^ AS-IV	429.1 ± 16.9	487.4 ± 19.4	485.2 ± 18.6	18.6 ± 0.23	17.5 ± 0.23	17.3 ± 0.24
DN-6 mg·kg^−1^ AS-IV	413.7 ± 11.8	504.3 ± 17.9	476.5 ± 21.5	18.6 ± 0.31	17.6 ± 0.28	17.7 ± 0.35
DN-12 mg·kg^−1^ AS-IV	428.1 ± 25.8	498.3 ± 19.3	481.6 ± 21.9	18.5 ± 0.38	18.0 ± 0.31	18.1 ± 0.44

8-week-old C57BL/6 J mice were injected intraperitoneally with 100 mg·kg^−1^ STZ for 2 consecutive days in the first week (week 0). 1 week after STZ injection (week 1), the mice were treated with AS-IV at the indicated doses of AS-IV for 8 consecutive weeks. Data are expressed as mean ± SEM. n = 8–12 per group. ^**^ *P* < 0.01 compared to NC-vehicle group. One-way ANOVA and Newman-Keuls multiple comparisons test. NC, nondiabetic control. DN, diabetic nephropathy.

Histological examination showed that DN-vehicle mice displayed larger glomerular size, an obvious mesangial matrix expansion, and severe glomerulosclerosis at week 9 compared with NC-vehicle mice. 12 mg^−1^·kg^−1^ day^−1^ AS-IV treatment markedly attenuated glomerular hypertrophy, mesangial matrix expansion and glomerulosclerosis in DN mice (Fig. 1D,E; Supplementary Fig. S1). The renin–angiotensin system (RAS) has been suggested as a major mediator of glomerular injury in DN^32^. We found that intrarenal RAS activity was upregulated in DN mice, as evidenced by elevated intrarenal Renin and angiotensinogen (AGT) mRNA expression and AGT protein expression, which was reversed by 12 mg^−1^·kg^−1^ day^−1^ AS-IV treatment (Supplementary Fig. S1). Inflammation is implicated in DN^33^. DN-vehicle mice exhibited intense renal inflammation, as indicated by immunohistochemistry staining and quantitative RT-PCR (qRT-PCR) detection of monocyte chemotactic protein-1 (MCP-1) and tumor necrosis factor-α (TNF-α), two inflammatory cytokines. However, AS-IV dose-dependently suppressed diabetes-induced MCP-1 and TNF-α upregulation (Supplementary Fig. S1).

Podocyte injury plays a key role in the development of albuminuria in diabetic nephropathy^3^. The number of apoptotic cells within the glomeruli significantly increased and podocyte number dramatically decreased in DN-vehicle mice compared with NC-vehicle mice, which was partially rescued by AS-IV administration, as evidenced by TUNEL staining and the podocyte nucleus marker Wilms’ tumor 1 (WT1) labeling (Fig. 1F–H). Podocyte foot process markers Podocin and Nephrin were also downregulated in DN-vehicle mice, as confirmed by immunofluorescence staining and western blot, indicating a damaged glomerular filtration barrier. However, AS-IV restored the expression of Podocin and Nephrin in a dose-dependent manner (Fig. 1F–J).

### AS-IV alleviated ER stress in STZ-induced diabetic mice

**Figure 2:**
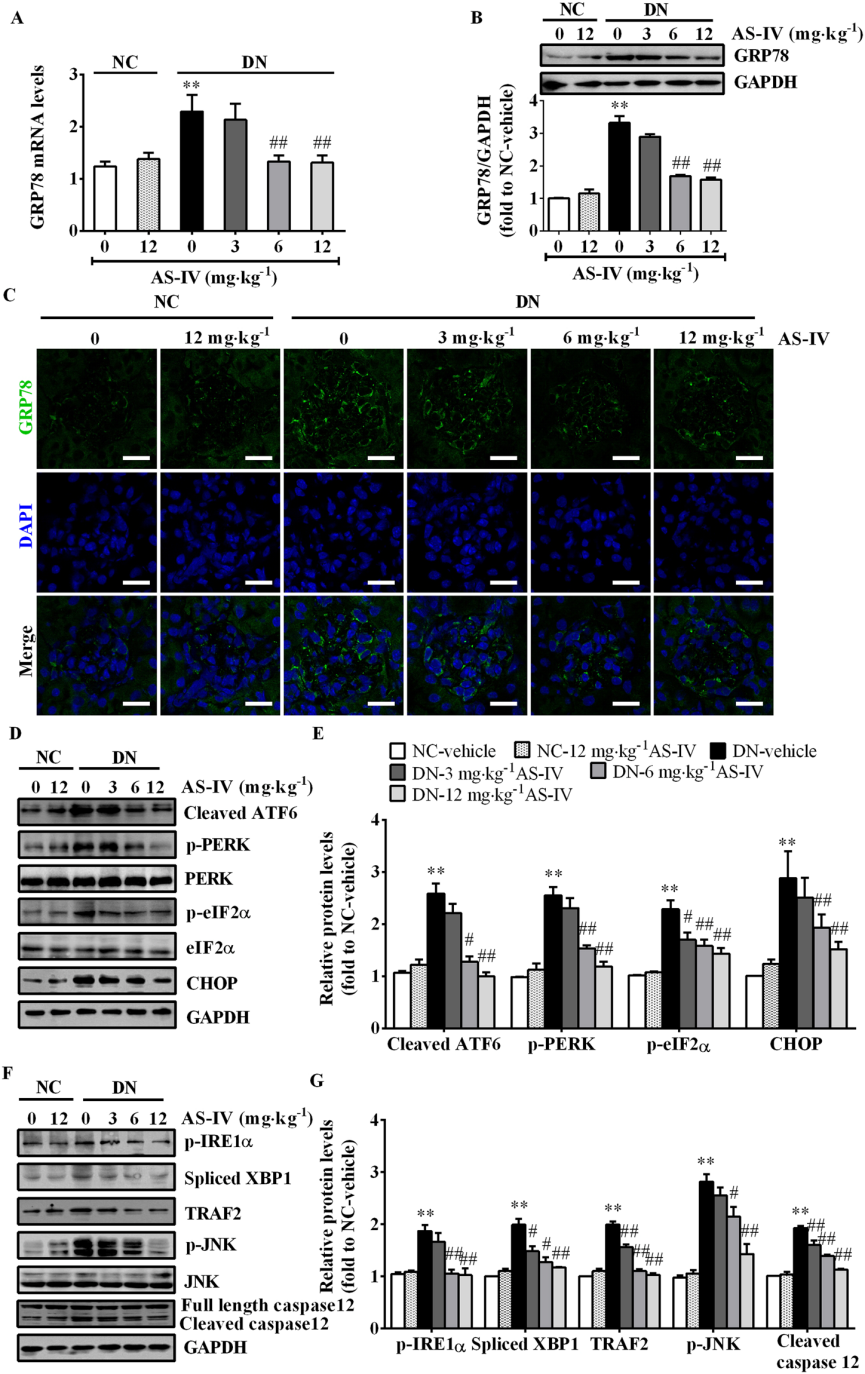
AS-IV repressed ER stress and ER stress-mediated apoptotic pathway in the kidney cortex of STZ-induced diabetic mice. (**A**) qRT-PCR analysis of GRP78 mRNA levels in each group. (**B**) Western blot analysis of GRP78 protein expression in the kidney cortex lysates from each group. (**C**) Immunofluorescence staining for GRP78 expression in each group. Scale bars, 20 μm. (**D**,**E**) Western blot analyses (**D**) and densitometric quantification (**E**) of cleaved ATF6, p-PERK, p-eIF2α and CHOP expression in total lysates of kidney cortex from each group. (**F** and **G**) Representative immunoblots (**F**) and densitometric quantification (**G**) of p-IRE1α, spliced XBP1, TNF receptor associated factor 2 (TRAF2), p-JNK and caspase 12 expression in total lysates of kidney cortex from each group. The original blots for panel B, D and F were presented in Supplementary Figure S3. Data are expressed as mean ± SEM. n = 4–6 per group. ^*^ *P* < 0.05, ^**^ *P* < 0.01 compared to NC-vehicle group; ^*#*^ *P* < 0.05, ^*##*^ *P* < 0.01 compared to DN-vehicle group. One-way ANOVA and Newman-Keuls multiple comparisons test (**A**,**B**,**E**,**G**). NC, non-diabetic control. DN, diabetic nephropathy.

Next, we investigated if the ER stress pathway was activated. As shown in Fig. 2A–C, compared with NC-vehicle mice, there were significant increases in both mRNA and protein levels of GRP78, an ER stress marker primarily localized to the glomeruli, in DN-vehicle mice, which were abrogated by AS-IV in a dose-dependent manner (Fig. 2A–C). Diabetes-induced activation of ER stress signaling transducers ATF6 and PERK, and their downstream targets, including phospho-eukaryotic translation initiation factor 2α (p-eIF2α) and pro-apoptotic transcription factor CHOP were significantly attenuated by AS-IV treatment (Fig. 2D,E). Likewise, AS-IV markedly reduced diabetes-induced phosphorylation of IRE1α and its downstream target JNK, an apoptosis mediator, and decreased spliced X-box binding protein 1 (XBP1) expression (Fig. 2F,G). In addition, the apoptosis marker protein cleaved caspase 12 was upregulated in DN mice but was attenuated by AS-IV (Fig. 2F,G). These findings supported that AS-IV mitigated ER stress and ER stress-induced apoptosis.

### AS-IV restored SERCA2b expression and activity in STZ-induced diabetic mice

**Figure 3:**
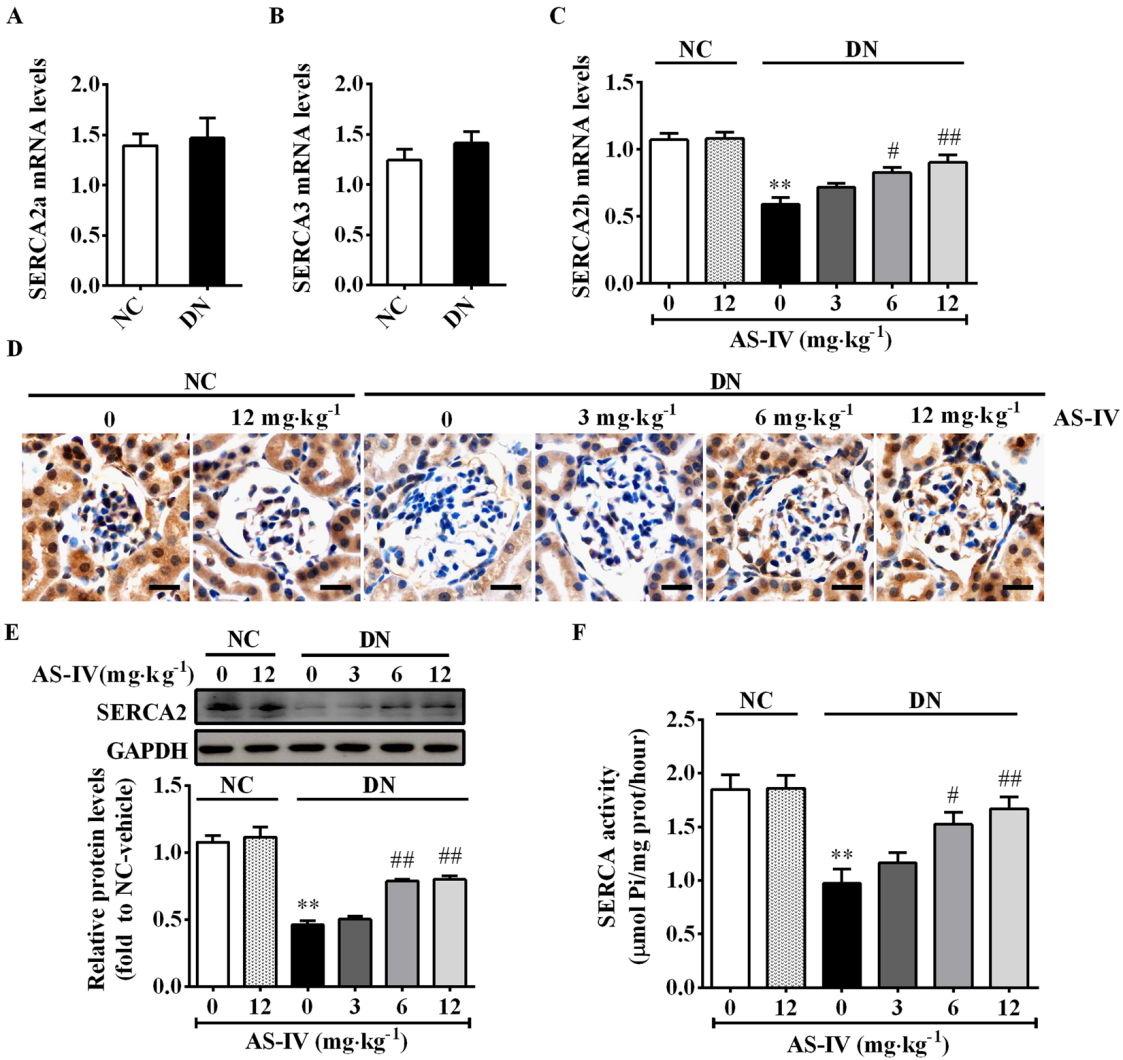
AS-IV restored the impaired expression and activity of SERCA2 in the kidney cortex of STZ-induced diabetic mice. (**A**) qRT-PCR analysis of SERCA2a mRNA expression in the renal cortex from NC group and DN group. (**B**) qRT-PCR analysis of SERCA3 mRNA expression in the renal cortex from NC group and DN group. (**C**) qRT-PCR analysis of SERCA2b mRNA expression in the renal cortex from each group. (**D**) Immunohistochemistry staining for SERCA2 protein expression in each group. Scale bars, 10 μm. (**E**) Western blot analysis and densitometric quantification of SERCA2 protein levels in the lysates of kidney cortex from each group. The original blots were presented in Supplementary Figure S4. (**F**) Relative SERCA2 activity in ER extraction of the kidney from each group. Data are expressed as mean ± SEM. n = 4–6 per group. ^**^ *P* < 0.01 compared to NC-vehicle group; ^*#*^ *P* < 0.05, ^*##*^ *P* < 0.01 compared to DN-vehicle group. Student’s *t* -test (**A**,**B**), One-way ANOVA and Newman-Keuls multiple comparisons test (**C**,**E**,**F**). NC, non-diabetic control. DN, diabetic nephropathy.

Dysfunction of SERCA has emerged as a major trigger for ER stress^34^. Our previous work has identified that SERCA2b was the predominant SERCA2b isoform expressed in the kidney and conditionally immortalized mouse podocyte cell line, while both SERCA2a and SERCA3 were expressed at extremely low levels. SERCA1 expression could not be detected^35^. Here, qRT-PCR analyses showed that SERCA2a and SERCA3 mRNA expression did not change significantly between DN mice and NC mice (Fig. 3A,B). SERCA2b mRNA and protein expression were dramatically downregulated in DN group compared with NC group, both of which were rescued by AS-IV in a dose-dependent manner (Fig. 3C–E). In parallel to a marked decrease in SERCA2 expression, SERCA activity almost decreased by 50% in DN mouse kidney compared to NC mice. However, treatment with AS-IV at 6 mg·kg^−1^·day^−1^ and 12 mg·kg^−1^·day^−1^ remarkably restored SERCA activity in the kidneys of DN mice (Fig. 3F).

### AS-IV restored the defective autophagy in STZ-induced diabetic mice

**Figure 4:**
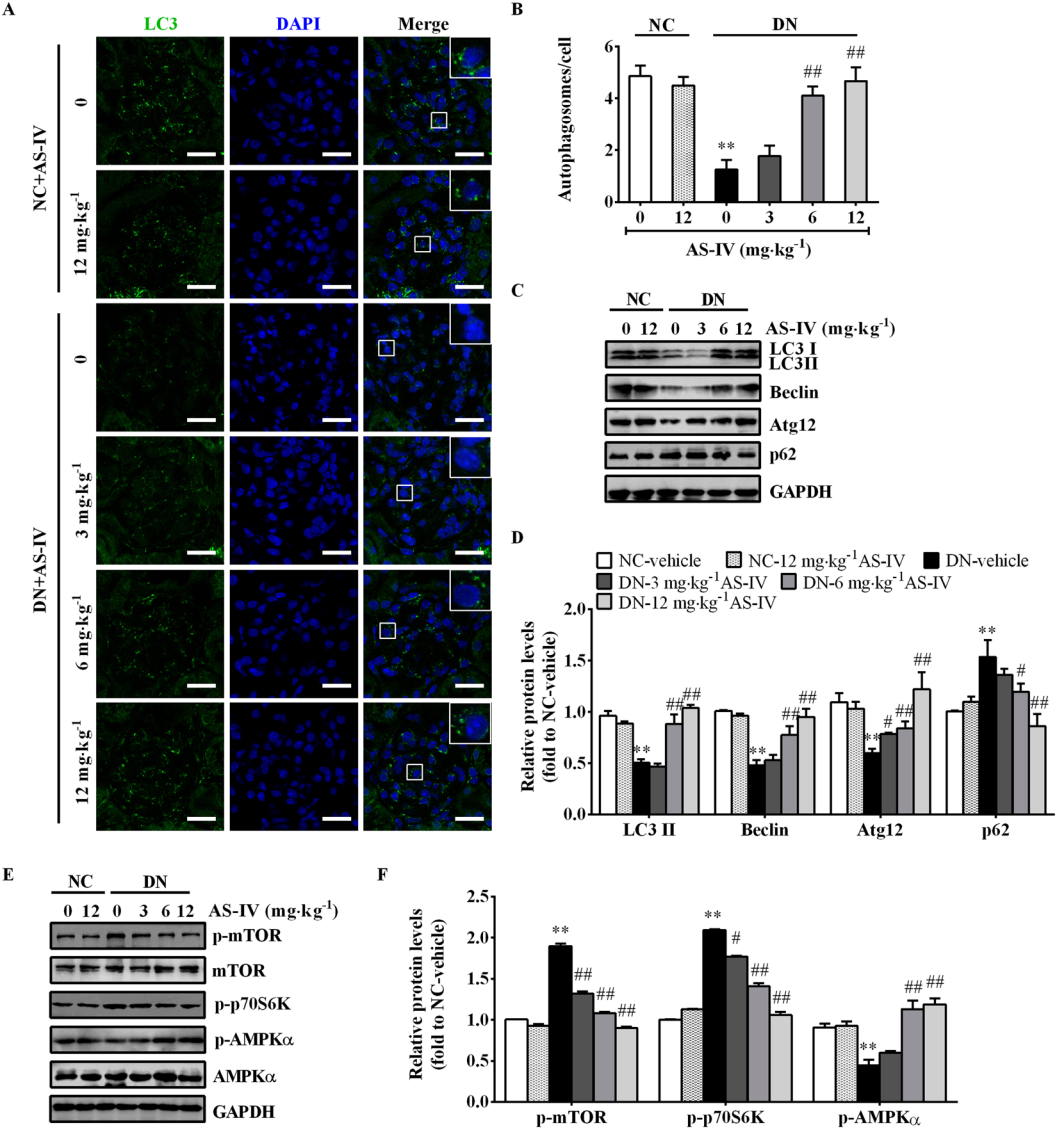
AS-IV repaired the defective autophagy in the glomeruli of STZ-induced diabetic mice. (**A**) Immunofluorescence staining for LC3B to examine autophagy activity in each group. Scale bars, 20 μm. (**B**) Quantitative analysis of LC3-labeled autophagosomes per cell in the glomeruli of each group. (**C**, **D**) Western blot analyses (**C**) and densitometric quantification (**D**) of autophagy-related proteins such as LC3, Atg12, Beclin and p62 in the kidney cortex lysates from each group. (**E**,**F**) Western blot analyses (**E**) and densitometric quantification (**F**) of p-mTOR, p-p70S6K and p-AMPKα expression in total lysates of kidney cortex from each group. The original blots for panel C and E were presented in Supplementary Figure S5. Data are expressed as mean ± SEM. n = 4–6 per group. ^**^ *P* < 0.01 compared to NC-vehicle group; ^*#*^ *P* < 0.05, ^*##*^ *P* < 0.01 compared to DN-vehicle group. One-way ANOVA and Newman-Keuls multiple comparisons test (**B**,**D**,**F**). NC, non-diabetic control. DN, diabetic nephropathy.

Autophagy is an adaptive response in cells under environmental stress and participates in the renal diseases^18^. To investigate the role of autophagy in DN, we examined the alterations of autophagy in DN by immunofluorescence staining of LC3. As shown in Fig. 4A, glomeruli exhibited high levels of autophagy activity under basal conditions while tubuli had extremely low levels of autophagy activity. At 9 weeks after STZ injection, autophagy activity was suppressed. However, AS-IV treatment could dose-dependently enhance the glomerular autophagy activity as evidenced by improved autophgosome formation (Fig. 4A,B). Concomitant with the immunofluorescence staining alterations, western blot analysis showed that the expression of autophagy-related proteins such as LC3A/B, Beclin-1 and Atg12 was markedly decreased and p62, a specific target of the autophagy degradation, was increased in kidney tissues at 9 weeks post-STZ injection, indicating a suppressed autophagy level. However, AS-IV treatment for 8 weeks largely reversed the expression of these components of the autophagy pathway (Fig. 4C,D). Moreover, we found that DN-vehicle mice displayed enhanced activation of mTOR signaling and impaired AMPK activity, as indicated by increased phosphorylation of mTOR and p70S6 kinase (p70S6K) and decreased phosphorylation of AMPKα. However, this alternation was prevented by AS-IV treatment (Fig. 4E,F). The above results indicated that AS-IV was able to induce autophagy possibly via activating AMPK in the kidneys of DN mice.

### AS-IV protected against HG-induced podocyte apoptosis

**Figure 5:**
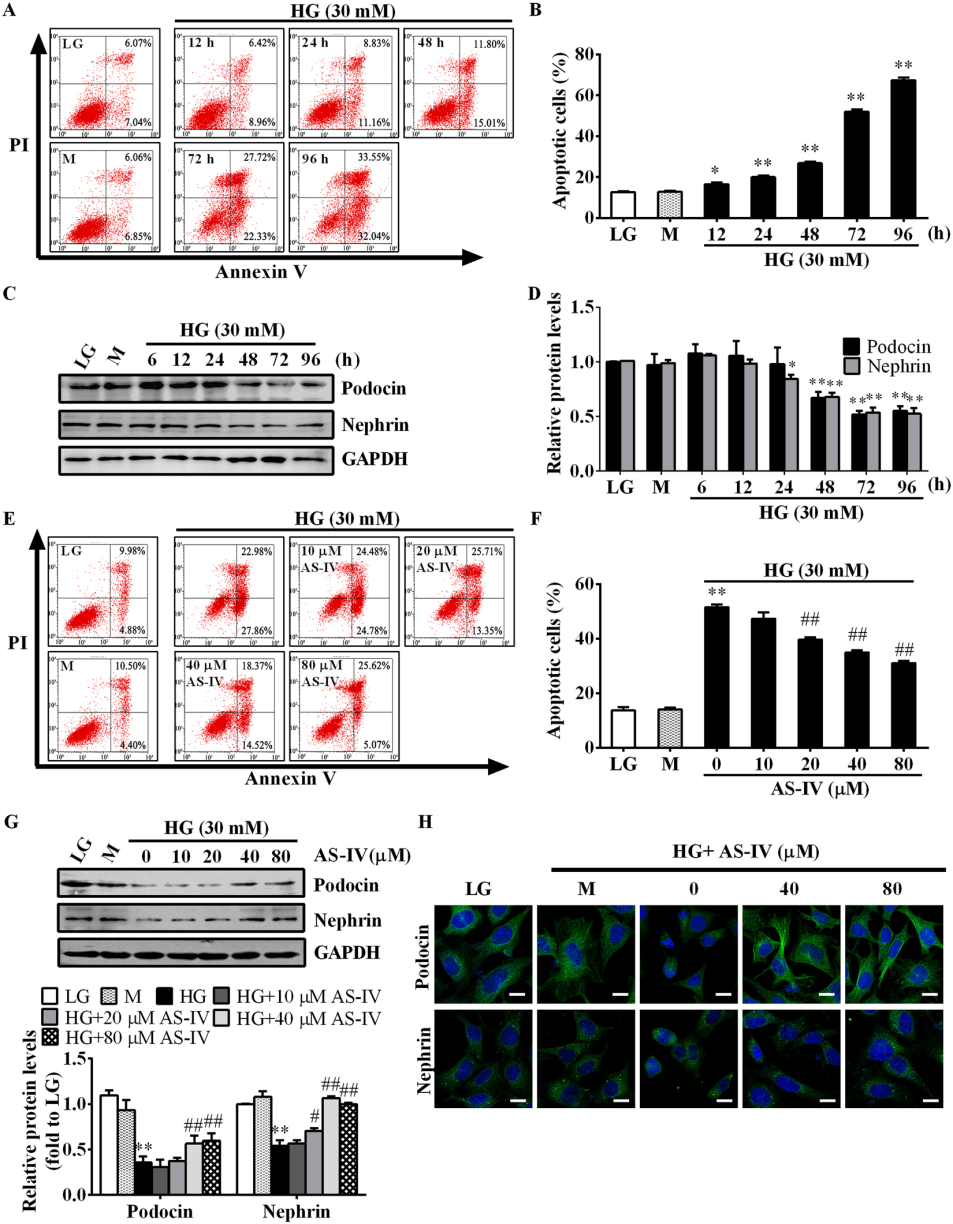
AS-IV reduced HG-induced podocyte apoptosis. (**A**–**D**) Podocytes were treated with 30 mM HG for 6, 12, 24, 48, 72 and 96 h. LG and M were used as controls. Podocytes were collected at different time points and subjected to analyses by flow cytometry or western blot. (**A**) Representative flow cytometry results for podocytes exposed to HG for various time periods as indicated. (**B**) Quantitative analysis of apoptotic cells determined by flow cytometry. (**C**,**D**) Western blot analyses (**C**) and densitometric quantification (**D**) of Podocin and Nephrin expression at various time points. Data are expressed as mean ± SEM. n = 3. ^*^ *P* < 0.05, ^**^ *P* < 0.01 compared to LG group. (**E**–**H**) Podocytes were pretreated with or without AS-IV at the indicated concentrations for 2 h followed by HG exposure for 72 h and then subjected to analyses by flow cytometry, western blot and immunofluorescence staining. (**E**,**F**) Representative flow cytometry pictures (**E**) and quantitative analysis (**F**) of apoptotic podocytes under different cultural conditions. (**G**) Western blot analyses and densitometric quantification of Podocin and Nephrin expression under different cultural conditions. (**H**) Immunofluorescence staining for Podocin and Nephrin under different cultural conditions. It revealed crimpled Podocin around the nuclei and reduced Nephrin expression. Scale bars, 10 μm. The original blots for panel C and G were presented in Supplementary Figure S6. Data are expressed as mean ± SEM. n = 3. ^**^ *P* < 0.01 compared to LG group. ^*#*^ *P* < 0.05, ^*##*^ *P* < 0.01 compared to HG group. One-way ANOVA and Newman-Keuls multiple comparisons test (**B**,**D**,**F**,**G**). LG, low glucose; M, mannitol; HG, high glucose.

Immortalized mouse podocytes were cultured in low glucose (LG), mannitol (M) or HG medium and the effect of high glucose on podocyte apoptosis was monitored. Flow cytometry analysis using Annexin V-FITC/PI staining revealed that HG induced apoptosis in a time-dependent manner (Fig. 5A,B), accompanied by a decline in the expression of podocyte markers Podocin and Nephrin as determined by western blot (Fig. 5C,D). Pretreatment with AS-IV attenuated HG-induced podocyte apoptosis in a concentration-dependent manner (Fig. 5E,F), paralleled by the restoration of podocyte Podocin and Nephrin as confirmed by western blot and immunofluorescence staining (Fig. 5G,H).

### AS-IV attenuated HG-triggered ER stress in a SERCA2-dependent mananer in podocytes

**Figure 6:**
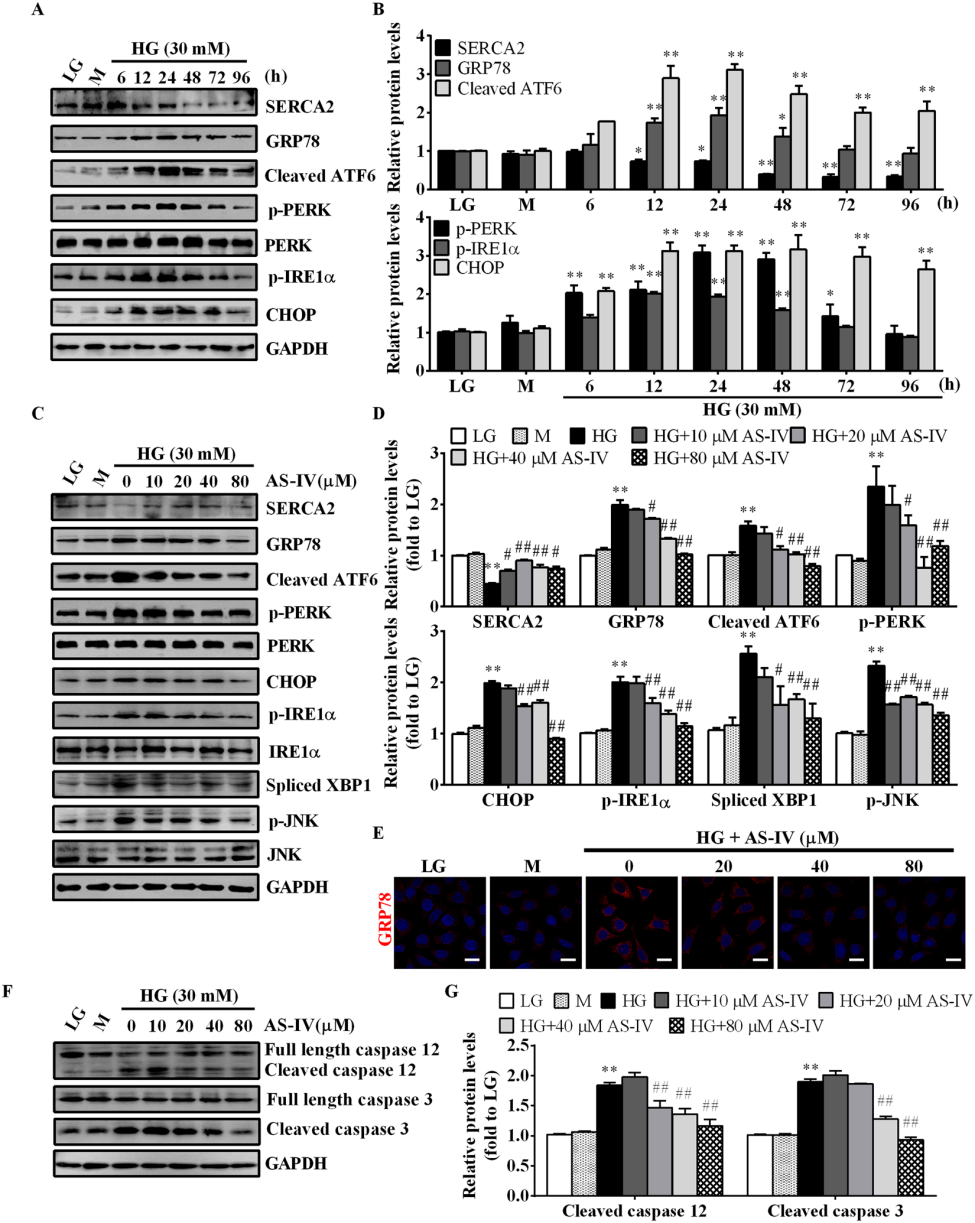
AS-IV mitigated HG-triggered ER stress in podocytes. (**A**,**B**) Podocytes were treated with 30 mM HG for 6, 12, 24, 48, 72 and 96 h. LG and M were used as controls. Western blot analyses (**A**) and densitometric quantification (**B**) of SERCA2 and ER stress-associated protein expression at the indicated time points. n = 3. ^*^ *P* < 0.05, ^**^ *P* < 0.01 compared to LG group. (**C**–**G**) Podocytes were pretreated with or without AS-IV at the indicated concentrations for 2 h followed by HG exposure for 24 h. LG and M groups were used as controls. (**C**,**D**) Western blot analyses (**C**) and densitometric quantification (**D**) of SERCA2 and ER stress markers under different cultural conditions. (**E**) Immunofluorescence staining for GRP78 at 24 h after HG incubation. Scale bars, 20 μm. (**F**,**G**) Representative immunoblots (**F**) and densitometric quantification (**G**) of apoptosis markers under different cultural conditions. The original blots for panel A, C and F were presented in Supplementary Figure S7. Data are expressed as mean ± SEM. n = 3. ^**^ *P* < 0.01 compared to LG group. ^*#*^ *P* < 0.05, ^*##*^ *P* < 0.01 compared to HG group. One-way ANOVA and Newman-Keuls multiple comparisons test (**B**,**D**,**G**). LG, low glucose; M, mannitol; HG, high glucose.

To determine whether ER stress was involved in HG-induced podocyte apoptosis, ER stress-related proteins were detected by western blot at various time points. Incubation with HG for 12–24 h significantly decreased the expression of SERCA2 and upregulated ER stress-related proteins such as GRP78, cleaved ATF6, p-PERK, p-IRE1 and CHOP, which indicated a strong induction of ER stress (Fig. 6A,B). However, pre-incubation of AS-IV improved HG-decreased SERCA2 expression and reversed the expression of HG-induced ER stress-associated proteins at 24 h post-HG stimulation in a concentration-dependent manner (Fig. 6C,D). Immunofluorescence staining of GRP78 also confirmed the repression of HG-induced ER stress by AS-IV (Fig. 6E). Additionally, HG-elevated expression of apoptosis mediators, including CHOP, p-JNK, cleaved caspase 12 and caspase 3, was significantly downregulated by AS-IV (Fig. 6A–G). These data demonstrated that AS-IV ameliorated HG-induced podocytes apoptosis partially through mitigating ER stress-mediated apoptotic pathway.

**Figure 7:**
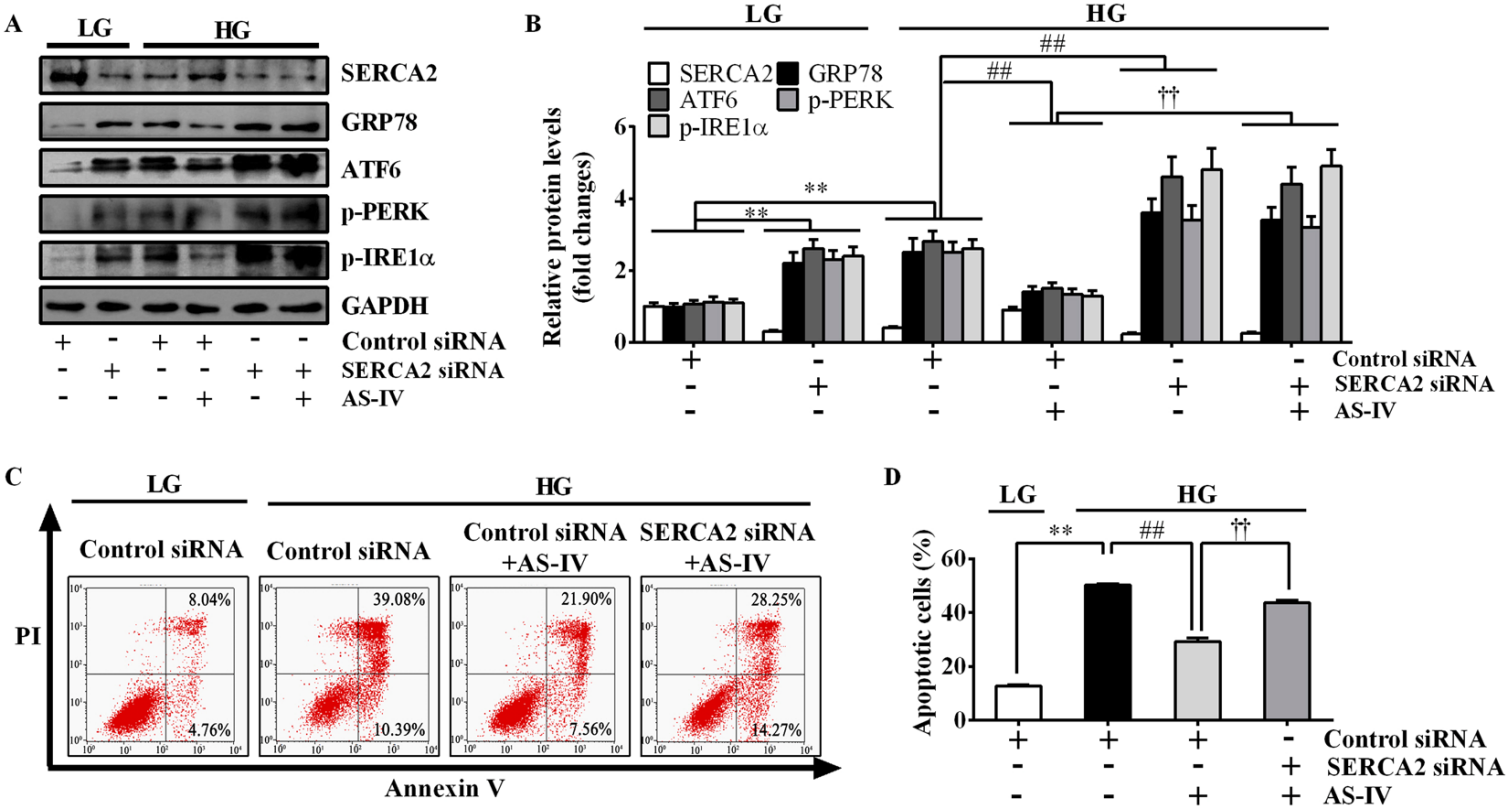
SERCA2 mediated the inhibitory effect of AS-IV on HG-triggered ER stress. Podocytes were transfected with control siRNA or specific SERCA2 siRNA for 6 h, and then treated with HG in the presence or absence of 80 μM AS-IV for 24 h (ER stress detection) or 72 h (apoptosis). (**A**) Representative immunoblots of SERCA2 and ER stress-associated proteins under different conditions as indicated. (**B**) Densitometric quantification of SERCA2 and ER stress-associated proteins as shown in panel A. (**C**) Representative flow cytometry results for podocytes cultured under different conditions as indicated. (**D**) Quantitative analysis of apoptotic cells determined by flow cytometry as shown in panel C. Data are expressed as mean ± SEM. n = 3. ^**^ *P* < 0.01 compared to LG plus control siRNA group. ^*##*^ *P* < 0.01 compared to HG plus control siRNA group. ^††^ *P* < 0.01 compared to HG plus control siRNA and AS-IV group. The original blots for panel A were presented in Supplementary Figure S8. One-way ANOVA and Newman-Keuls multiple comparisons test (**B**,**D**). LG, low glucose; HG, high glucose.

Since SERCA2 has been recognized as a key regulator of ER stress, we next investigated whether SERCA2 mediated the beneficial effect of AS-IV on ER stress in podocytes. As shown in Fig. 7A, transfection of SERCA2b-specific siRNA (MSS202247), but not control siRNA, significantly reduced SERCA2 expression in podocytes under basal condition. Gene silencing of SERCA2 markedly induced ER stress in podocytes under LG condition and enhanced HG-induced ER stress, suggesting that SERCA2b inhibition triggered ER stress in podocytes (Fig. 7A,B). In addition, SERCA2b silencing abrogated AS-IV-alleviated ER stress (Fig. 7A,B). Flow cytometry analysis showed that down regulating SERCA2 expression could significantly attenuated the anti-apoptotic effect of AS-IV (Fig. 7C,D). These data confirmed the critical role of SERCA2 in mediating the protective effect of AS-IV on HG-triggered ER stress and apoptosis.

### AS-IV restored HG-inhibited autophagy through AMPKα activation in podocytes

**Figure 8:**
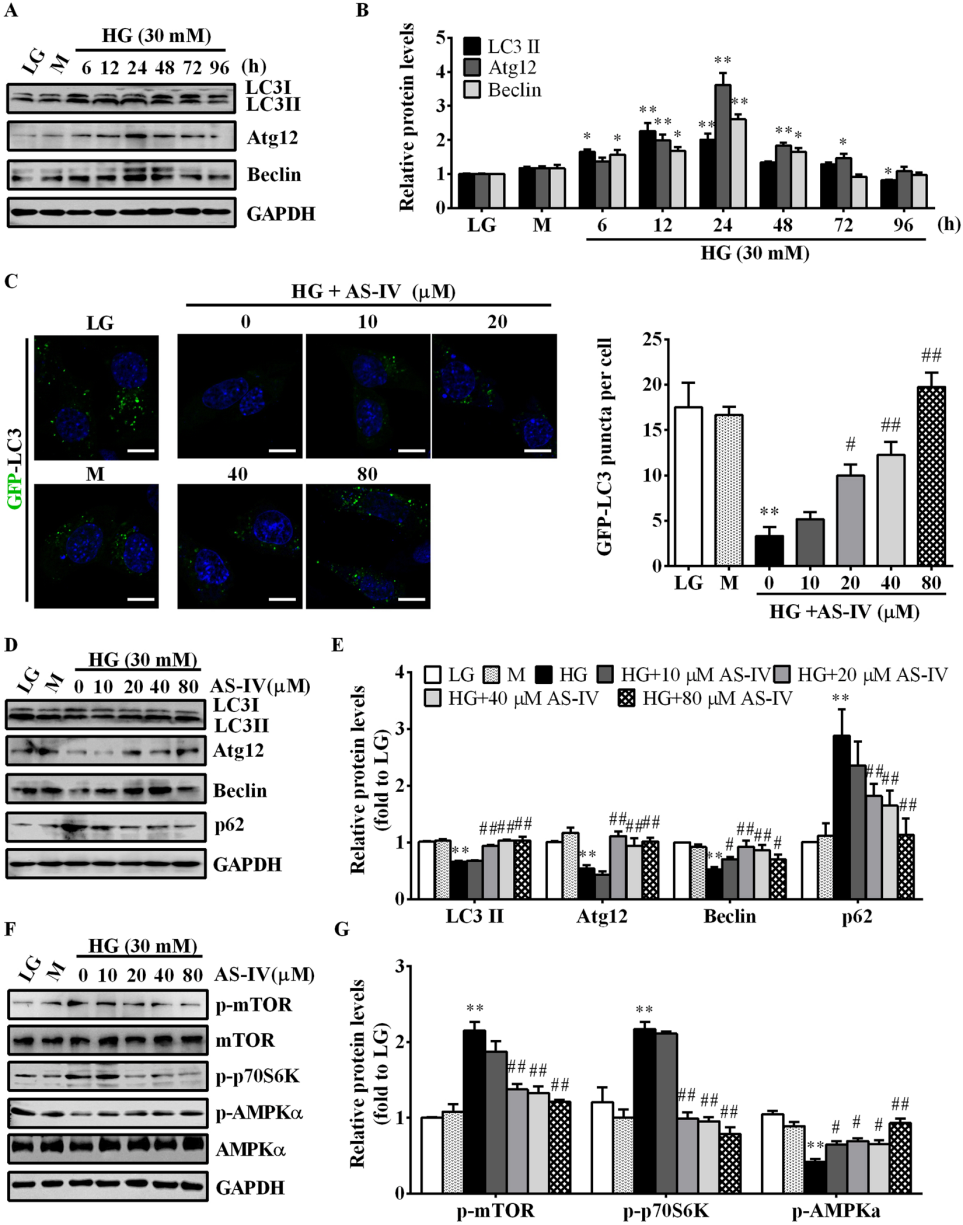
AS-IV improved HG-compromised autophagy activity in podocytes. (**A**,**B**) Podocytes were treated with 30 mM HG for 6, 12, 24, 48, 72 and 96 h. LG and M were used as controls. Western blot analyses (**A**) and densitometric quantification (**B**) of autophagy-related protein expression at various time points. Data are expressed as mean ± SEM. n = 3. ^*^ *P* < 0.05, ^**^ *P* < 0.01 compared to LG group. (**C**–**G**) Podocytes were pretreated with or without AS-IV at the indicated concentrations for 2 h followed by GFP-LC3 expression plasmid transfection and HG exposure for 72 h. (**C**) Representative images showing the autophagic flux after AS-IV treatment and quantification of GFP-LC3 puncta per cell in each group. The appearance of dot-like GFP-LC3 puncta indicated autophagosome formation. Scale bars, 10 μm. (**D**,**E**) Western blot analyses (**D**) and densitometric quantification (**E**) of LC3A/B, Atg12, Beclin and p62 expression in each group. (**F**,**G**) Western blot analyses (**F**) and densitometric quantification (**G**) of p-mTOR, p-p70S6K and p-AMPKα expression in each group. The original blots for panel A, D and F were presented in Supplementary Figure S9. Data are expressed as mean ± SEM. n = 3. ^**^ *P* < 0.01 compared to LG group. ^*#*^ *P* < 0.05, ^*##*^ *P* < 0.01 compared to HG group. One-way ANOVA and Newman-Keuls multiple comparisons test (**B**,**E**,**G**). LG, low glucose; M, mannitol; HG, high glucose.

**Figure 9:**
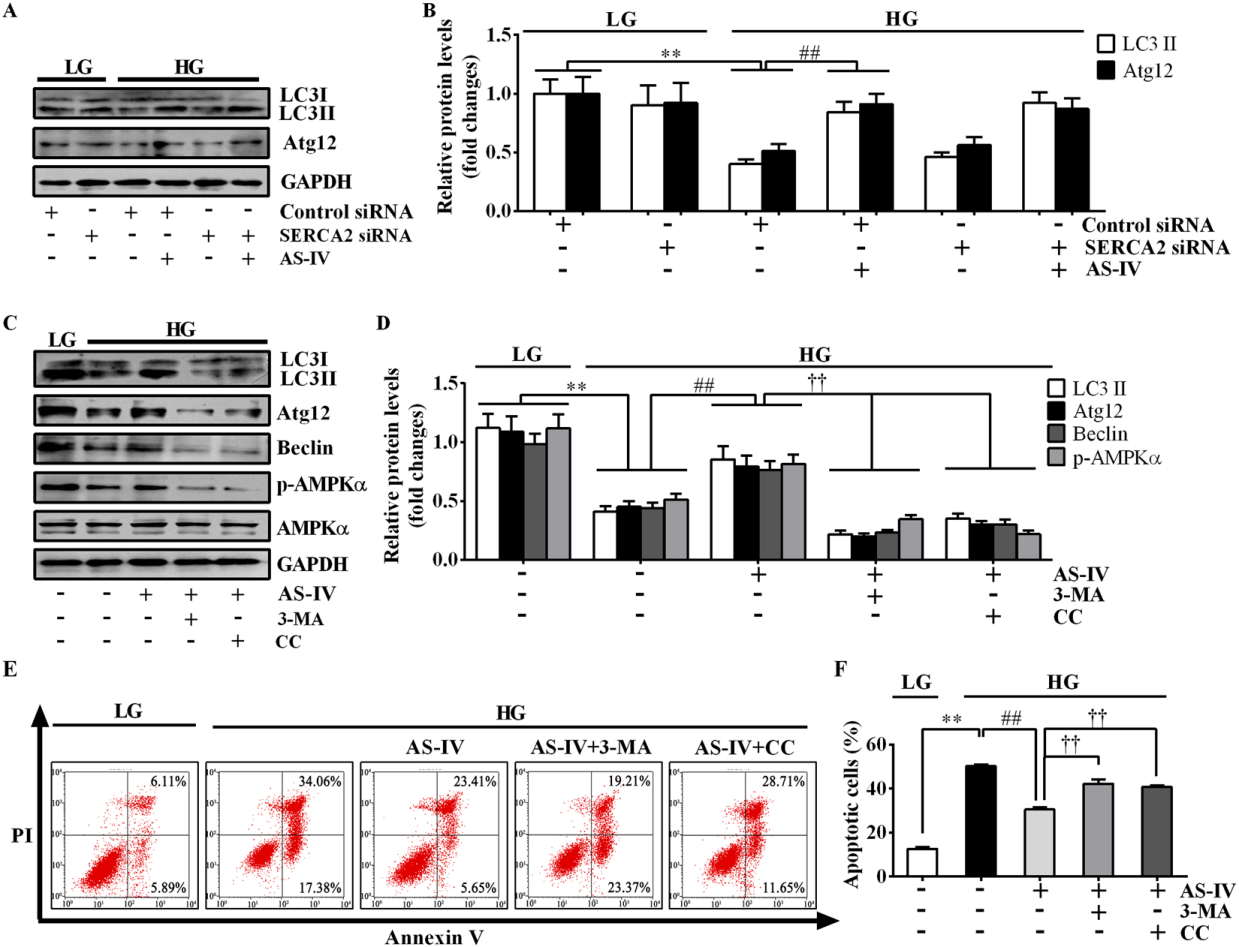
AMPKα mediated the beneficial effect of AS-IV on HG-induced autophagy defect. (**A**,**B**) Podocytes were transfected with control siRNA or specific SERCA2 siRNA for 6 h, and then treated with HG in the presence or absence of 80 μM AS-IV for 72 h. (**A**) Representative immunoblots of LC3 and Atg12 under different conditions as indicated. (**B**) Densitometric quantification of LC3-II and Atg12 as shown in panel A. Data are expressed as mean ± SEM. n = 3. ^**^ *P* < 0.01 compared to LG plus control siRNA group. ^*##*^ *P* < 0.01 compared to HG plus control siRNA group. (**C**–**F**) Podocytes were treated with HG in the presence or absence of AS-IV (80 μM) or the autophagy inhibitor 3-MA (1 mM) or the AMPK inhibitor CC (1 μM) for 72 h. (**C**) Representative immunoblots of LC3, Atg12, Beclin and p-AMPKα under different conditions as indicated. (**D**) Densitometric quantification of LC3, Atg12, Beclin and p-AMPKα as shown in panel C. (**E**) Representative flow cytometry results for podocytes cultured under different conditions as indicated. (**F**) Quantitative analysis of apoptotic cells determined by flow cytometry as shown in panel E. Data are expressed as mean ± SEM. n = 3. ^**^ *P* < 0.01 compared to LG group. ^*##*^ *P* < 0.01 compared to HG group. ^††^ *P* < 0.01 compared to HG plus AS-IV group. The original blots for panel A and C were presented in Supplementary Figure S10. One-way ANOVA and Newman-Keuls multiple comparisons test (**B**,**D**,**F**). LG, low glucose; HG, high glucose; 3-MA, 3-methyladenine; CC, compound C.

To delineate the role of autophagy in HG-stimulated podocytes, we examined the expression pattern of autophagy-related proteins after HG stimulation. HG incubation for 12–24 h remarkably induced the expression of autophagy-related proteins, which then began to decline after treatment with HG for 48 h (Fig. 8A,B). This observation confirmed a short-term inductive effect of HG on autophagy and a long-term suppressive effect of HG on autophagy in podocytes. The transfection of fluorescent reporter GFP-LC3 to monitor autophagic flux in podocytes also validated defective autophagy at 72 h post-HG incubation, while AS-IV could restore the insufficient autophagy in a concentration-dependent manner as indicated by the increased number of dot-like GFP-LC3 puncta (Fig. 8C). Western blot analysis showed that AS-IV increased HG-diminished abundance of LC3A/B, Atg12 and Beclin, and reduced the accumulation of p62 (Fig. 8D,E). In addition, HG stimulation for 72 h markedly enhanced the phosphorylation of mTOR and p70S6K and reduced the phosphorylation of AMPKα, which were also reversed by AS-IV in a concentration-dependent manner (Fig. 8F,G). While SERCA2b silencing did not alter the expression of autophagy markers in HG-treated podocytes (Fig. 9A,B), autophagy inhibitor 3-methyladenine (3-MA) or AMPKα inhibitor compound C (CC) markedly antagonized the beneficial effect of AS-IV on autophagy induction and HG-induced apoptosis (Fig. 9C–F), suggesting the crucial role of AMPKα-promoted autophagy induction in mediating the protective effect of AS-IV in HG-induced podocytes and that SERCA2 is not required for autophagy induction.

## Discussion

In the present study, we used a STZ-induced diabetic model to investigate the effect of AS-IV on DN progression and gained further insight into the mechanisms underlying the beneficial action of AS-IV *in vivo* and *in vitro*. We demonstrated that AS-IV exhibited a prominent renoprotection in DN development, as evidenced by enhanced renal function, ameliorated glomerulosclerosis and renal inflammation. The protective effect of AS-IV was associated with ER stress attenuation via SERCA2b restoration and autophagy enhancement through AMPKα activation, which subsequently protected podocytes from hyperglycemia or HG-induced apoptosis. This investigation highlights AS-IV as an antiproteinuric drug targeting podocytes for DN therapy.

DN is a long-term complication of diabetes that develops in about 30% of patients with type 1 and 10% of patients with type 2 diabetes, with proteinuria and glomerulosclerosis as the pathological hallmarks^3, 36^. Microalbuminuria is the earliest sign and a major risk factor for progressive renal function decline in DN^37^. In our study, STZ-treated mice developed progressive albuminuria and elevated serum BUN and creatinine, as well as significant glomerular hypertrophy, mesangial matrix expansion and glomerulosclerosis similar to human DN, indicating the impaired renal function and the development of DN in our animal model. Interestingly, AS-IV treatment markedly attenuated albuminuria, reduced serum BUN and creatinine, and ameliorated glomerular histopathology and inflammation, suggesting AS-IV effectively prevented the progression of DN. However, AS-IV treatment had no obvious effects on body weight and fasting blood glucose, which is consistent with the investigation by Chen *et al*.^30^. Our previous work conducted in *db/db* mice, a mouse model for type 2 diabetes, showed that AS-IV could decrease hyperglycemia^35^. In the present study, AS-IV could not lower hyperglycemia in the type 1 diabetes model, which indicated that the improved renal function might be a direct effect of AS-IV on podocytes. As expected, hyperglycemia or HG-induced podocyte apoptosis was significantly attenuated by AS-IV, confirming the protective effect of AS-IV on podocytes. Glomerular hypertrophy is one of the earliest pathological alterations found in DN^38^. To further elucidate the mechanism underlying AS-IV-mediated amelioration of diabetes-induced glomerular hypertrophy despite the unaffected glycemic control, we examined renal components of RAS given the key role of the intrarenal RAS in DN development, including increasing glomerular capillary pressure and permeability, stimulation of renal cell proliferation and hypertrophy, and synthesis of cytokines and extracellular matrix^39^. We found that intrarenal Renin and AGT mRNA levels as well as AGT protein levels were markedly induced in DN group and downregulated after AS-IV treatment. Thus, our study suggested that the inhibtion of renal RAS activation was most likely the basis for AS-IV-suppressed glomerular hypertrophy in DN.

SERCA, an imperative maintainer for ER Ca^2+^ ^9^, is a major regulator of ER stress. Emerging evidence reveal that the activity or expression of SERCA was compromised in metabolic syndromes, resulting in ER stress and apoptosis, while the restoration of SERCA2 activity or expression ameliorated ER stress and in turn improved metabolic abnormalities^14, 40^. In our study, both SERCA2 expression and activity were significantly decreased in renal cortex of DN mice and HG-incubated mouse podocytes, paralleled by a marked induction of ER stress and the activation of both UPR signaling pathway and ER stress-mediated apoptotic pathway. AS-IV significantly restored hyperglycemia or HG-impaired expression of SERCA2 with a concomitant remission of ER stress. The data are in agreement with the report that AS-IV has pharmacological activities against podocyte injury through ER stress inhibition^30^. Furthermore, SERCA2 knockdown dramatically triggered ER stress and abolished AS-IV-reduced ER stress in podocytes, indicating SERCA2 mediated the inhibitory effect of AS-IV on HG-induced ER stress. Our results suggest that AS-IV can protect diabetes/HG-inflicted podocyte apoptosis at least in part via an ER stress-inhibiting mechanism involving SERCA2.

Though several investigations have been performed to define function of autophagy in the kidneys^20, 41, 42^, the role of autophagy in DN is still incompletely understood. There are some reports suggesting that autophagy is protective^22–24, 43^, while some indicate that autophagy might be pathogenic^44^. Our data showed that autophagy was expressed at high levels under basal conditions and compromised in 8-week-old diabetic mice, and short-term HG incubation induced autophagy while long-term HG incubation suppressed autophagy in podocytes *in vitro*. These observations were similar to other studies^22, 23, 45^. Interestingly, the deficient autophagy was restored by AS-IV in a dose-dependent manner. Autophagy is regulated by the nutrient-sensing pathways, including AMPK and mTOR^16^. AMPK activity decreases and mTORC1 activity increases in human and experimental type 1 and type 2 DN^20^. As expected, our study revealed a suppressed activity of AMPKα and an enhanced activity of mTOR in DN mice and HG-treated podocytes, which were significantly reversed by AS-IV. Either autophagy inhibitor 3-MA or AMPKα inhibitor CC could partly antagonize the protective effect of AS-IV against HG-induced autophagy defect and podocyte apoptosis, suggesting the crucial role of autophagy induction and AMPKα activation in mediating the anti-apoptotic effect of AS-IV in HG-treated podocytes. However, SERCA2 silencing had no obvious effect on AS-IV-improved autophagy activity, indicating autophagy repairment was not dependent on SERCA2-mediated ER stress reduction.

The UPR-associated proteins ATF6 and PERK have been reported to induce autophagy, while IRE1α acts a negative regulator of autophagy^46, 47^. Autophagy is activated during ER stress to supplement endoplasmic reticulum-associated degradation (ERAD), which mitigates ER stress and reduces cell death under conditions of ER stress^48, 49^. Autophagy inhibition can induce ER stress and podocyte apoptosis by activating the pro-apoptotic pathway^50^. Podocyte-specific deficiency of autophagy exacerbates proteinuria in diabetic nephropathy^24^ and leads to a glomerulopathy in aging mice that was related to ER stress^23^. Collectively, these findings suggest that autophagy represents an adaptive response of podocytes to ER stress that is renoprotective in DN. On the other hand, another study revealed that ER stress mediates HG-induced defective autophagy in podocytes^22^. The establishment of a causal relationship between autophagy and ER stress reduction needs further studies.

In conclusion, this study provides evidence that AS-IV protects against diabetes/HG-induced podocyte apoptosis by a mechanism involving ER stress attenuation mediated by SERCA2 restoration and autophagy enhancement promoted by AMPKα activation, which subsequently attenuates the albuminuria and prevents the progression of DN. This study highlights the role of SERCA and autophagy in the beneficial action of AS-IV on DN development, which may elicit a new therapeutic strategy for DN.

## Methods

### Animals and treatment

6-week old male C57BL/6J mice were purchased from Shanghai laboratory animal center (Shanghai, China). After 2 weeks of acclimation, the animals were induced to diabetes by intraperitoneal injection of freshly prepared STZ (Sigma-Aldrich, St Louis, MO, USA, dissolved in 0.01 M citrate buffer, pH 4.5) at 100 mg·kg^−1^·day^−1^ for 2 consecutive days. 1 week after STZ injection, fasting blood glucose was measured to verify the development of diabetes. Mice with blood glucose >350 mg·dL^−1^ were randomly separated into 4 groups and treated respectively with vehicle, 3 mg·kg^−1^ AS-IV, 6 mg·kg^−1^ AS-IV and 12 mg·kg^−1^ AS-IV by daily gavage for 8 weeks. NC mice without STZ treatment were randomly divided into 2 groups and administered respectively with vehicle and 12 mg·kg^−1^ AS-IV as controls. AS-IV was purchased from Shanghai Bogoo Biotechnology company, Limited (purity at 98%, Shanghai, China) and suspended in 0.5% carboxymethyl cellulose as a vehicle for the administration. All the work was carried out in accordance with the approved guidelines for the use of experimental animals in Putuo Hospital, Shanghai University of Traditional Chinese Medicine.

### Measurement of metabolic and biochemical parameters

Body weight, fasting glucose, and urine volume were measured at 4-week intervals. Fasting blood glucose was monitored with Omron HEA-230 Glucometer (Omron Corporation, Kyoto, Japan) by using one drop of tail blood. Animals were placed in individual metabolic cages every 4 weeks for 24 h urine collection and drinking volume calculation. Blood samples were drawn from orbit for serum BUN and creatinine detection at the end of the study. All mice were killed at 9 weeks after the start of STZ treatment, and kidneys were immediately harvested for protein or RNA extraction or for histological analysis. Urinary albumin and creatinine were measured with commercial ELISA kits (Biovision, Milpitas, CA, USA). Urinary albumin excretion was expressed as ACR. BUN was measured using Urea Nitrogen Colorimetric Detection Kit (Arbor Assays, Ann Arbor, MI, USA). All the biochemical parameters were determined according to the manufacturer’s instructions.

### Renal histology and immunohistochemistry

The kidneys were fixed in 4% paraformaldehyde (PFA), dehydrated, embedded in paraffin and cut into 5 μm-thick sections. Renal sections were stained with periodic acid-Schiff (PAS). Semiquantitative scoring of glomerular sclerosis was performed using a five-grade method described previously^51^. At least 50 glomeruli per section were evaluated by an examiner masked to the experimental conditions. For immunohistochemistry, paraffin-embedded sections were stained with primary antibodies against WT1 (ab89901, Abcam, Cambridge, MA, USA), MCP-1 (ab25124, Abcam, Cambridge, MA, USA), SERCA2 (ab2861, Abcam, Cambridge, MA, USA), and TNF-α (sc-52746, Santa Cruz Biotechnology, Santa Cruz, CA, USA) overnight at 4 °C. After incubation with biotinylated secondary antibody (Vector Laboratories, Burlingame, CA, USA), the sections were incubated with VECTASTAIN ABC reagent (Vector Laboratories, Burlingame, CA, USA) and color development was achieved using 3, 3′ diaminobenzidine (Vector Laboratories, Burlingame, CA, USA).

### TUNEL staining

The apoptotic cells in glomeruli were evaluated by TUNEL staining with the ApopTag Plus Peroxidase *In Situ* Apoptosis Detection Kit (Millipore, Billerica, MA, USA) according to the manufacturer’s instructions. TUNEL-positive cells were semiquantified by randomly counting 30 glomeruli in each mouse.

### Immunofluorescence staining

Kidneys were embedded in OCT compound by snap frozen in liquid nitrogen. The frozen sections (6 μm) were fixed with cold acetone for 10 min, blocked in PBS containing 5% goat serum at room temperature for 1 h, and incubated with primary antibodies against Podocin (ab50339, Abcam, Cambridge, MA, USA), Nephrin (ab58968, Abcam, Cambridge, MA, USA), LC3 (L7543, Sigma-Aldrich, St Louis, MO, USA), and glucose-regulated protein 78 (GRP78, ab21685, Abcam, Cambridge, MA, USA) at 4 °C overnight. After several PBS rinses, Cy^TM^ 2-conjugated secondary antibodies (Jackson ImmunoResearch, West Grove, PA, USA) were applied for 1 h at room temperature. Podocytes cultured on coverslips were fixed with 4% paraformaldehyde and permeabilized with 0.1% Triton X-100 for 5 min. Then cells were blocked with 5% BSA in PBS for 1 h at room temperature and incubated with the specific primary antibodies against Podocin (ab50339, Abcam, Cambridge, MA, USA), Nephrin (ab58968, Abcam, Cambridge, MA, USA), and GRP78 (ab21685, Abcam, Cambridge, MA, USA) followed by staining with Cy^TM^ 2 or Cy^TM^ 3-conjugated secondary antibodies (Jackson ImmunoResearch, West Grove, PA, USA). The nuclei were visualized by 4′,6-diamidino-2-phenylindole (DAPI) staining. Images were taken using a LEICA laser scan microscope (LEICA DM IRB; Leica, Wetzlar, Germany). The number of autophagosomes per cell in the glomeruli was quantified based on LC3-positive staining foci randomly selected from 20 glomeruli per mouse.

### Western blot analysis

Renal tissues or podocytes from each group were lysed with RIPA lysis buffer complemented with protease inhibitor cocktail and phosphatase inhibitor (both from Sigma-Aldrich, St Louis, MO, USA). Detection of protein expression by western blotting were performed as described previously^14^. Primary antibodies used for detection of SERCA2 (sc-8094), eIF2α (sc-133132), JNK (sc-571), p-JNK (Thr183/Tyr185, sc-6254) were purchased from Santa Cruz Biotechnology (Santa Cruz, CA, USA); for Podocin (ab50339), Nephrin (ab58968), GRP78 (ab21685), ATF6 (ab11909), XBP1(ab37152), and AMPKα (ab80039) from Abcam (Cambridge, MA, USA); for GAPDH (#2118), IRE1α (#3294), PERK (#3192), p-PERK (Thr980, #3179), p-eIF2α (Ser51, #9721), CHOP (#2895), caspase 12 (#2202), caspase 3 (#9665), LC3A/B (#12741), Beclin (#3738), Atg12 (#4180), p62 (#5114), mTOR (#2983), p-mTOR (Ser2448, #5536), p-AMPKα (Thr172, #2535), and p-p70S6K (Thr389, #9205) from Cell Signaling Technology (Danvers, MA, USA); for p-IRE1α (Ser724, NB100-2323) from Novus Biologicals (Littleton, CO, USA); for AGT (11992-1-AP) from Proteintech (Rosemont, IL, USA). Horseradish peroxidase–conjugated goat anti-rabbit IgG, goat anti-mouse IgG and rabbit anti-goat IgG were purchased from BOSTER (Wuhan, China). The blot images were produced by ImageQuant LAS 500 imaging system (GE Healthcare Bio-sciences AB, Uppsala, Sweden). Densitometric quantitation was performed using Image J 1.37 software (NIH, Bethesda, MD, USA). Protein expression was normalized with GAPDH and with total proteins for phosphorylated proteins.

### qRT-PCR

Total RNA was extracted from renal cortex using Trizol reagent (Invitrogen, Carlsbad, CA, USA). Then first strand cDNAs were synthesized from 1 μg of total RNA in a 20 μl reaction volume using Moloney murine leukemia virus reverse transcriptase (New England Biolabs, Ipswich, MA, USA) and hexanucleotide random primers (Takara, Dalian, China). qRT-PCR was performed in an Applied Biosystems ViiA™ 7 real time PCR system using SuperReal PreMix Plus (SYBR Green) Kit (TIANGEN BIOTECH (BEIJING) CO., LTD; Beijing, China). The primer sets used for qRT-PCR were listed in Table 2, among which equally efficient primers for SERCA2a, SERCA2b, SERCA3 and primers for the RAS genes were used as previously described^10, 52^. All the primers were synthesized by Sangon Biotech (Shanghai) Co., Ltd. (Shanghai, China). The reactions were prepared in triplicate and heated to 95 °C for 10 min, followed by 40 cycles at 95 °C for 15 s, and at 60 °C for 1 min. The relative mRNA amount was normalized to the invariant GAPDH mRNA.

**Table 2:** List of primers used for qRT-PCR.

	Forward primer (5′-3′)	Reverse primer (5′-3′)
SERCA2a	GATCCTCTACGTGGAACCTTTG	GGTAGATGTGTTGCTAACAACG
SERCA2b	GATCCTCTACGTGGAACCTTTG	CGACAGGGAGCAGGAAGAT
SERCA3	AGGGGAAGCTAAGAAGCCAG	CCCTCAGACTCCTCCTACCC
MCP-1	GCTCAGCCAGATGCAGTTAA	TCTTGAGCTTGGTGACAAAAACT
TNF-α	CATGAGCACAGAAAGCATGATCCG	AAGCAGGAATGAGAAGAGGCTGAG
GRP78	TTCAGCCAATTATCAGCAAACTCT	TTTTCTGATGTATCCTCTTCACCAGT
Renin	GAGGCCTTCCTTGACCAATC	TGTGAATCCCACAAGCAAGG
Angiotensinogen	ACGTTCACTTCCAAGGAACGA	TCACTCCAGTGCTGGAAGTT
GAPDH	TCACCACCATGGAGAAGGC	GCTAAGCAGTTGGTGGTGCA

### Cell culture and treatment

Mouse podocyte cell lines, kindly provided by Prof. Niansong Wang (Shanghai Sixth People’s Hospital, China) and originally provided by Dr. Peter Mundel (Division of Nephrology, Massachusetts General Hospital, Harvard University), were cultured as previously described^53^. The differentiated podocytes were pretreated with or without AS-IV (10, 20, 40 or 80 μM) for 2 h followed by treatment with low D-glucose (5 mM, LG), mannitol (25 mM mannitol +5 mM D-glucose, M) or high glucose (30 mM, HG) for 24 or 72 h. To determine whether SERCA2 was involved in the effect of AS-IV on ER stress, stealth siRNA for mouse SERCA2 (MSS202247, MSS202248, MSS202249) and control siRNA (Stealth RNAi Negative Control kit, Cat: 12935112) (both form Invitrogen, Carlsbad, CA, USA) were transfected with Lipofectamine^®^ RNAiMAX Transfection Reagent (Invitrogen, Carlsbad, CA, USA) for 6 h before being incubated with 30 mM HG medium with or without 80 μM AS-IV treatment. To evaluate the role of autophagy and AMPKα in mediating the function of AS-IV, podocytes were treated with HG in the presence or absence of AS-IV (80 μM) or the autophagy inhibitor 3-MA (1 mM, Cat: S2767, Selleckchem, Houston, TX, USA) or the AMPK inhibitor CC (1 μM, Cat: EY1783; AMQUAR Bio, CO, USA). AS-IV and CC were dissolved in DMSO and the final DMSO concentration did not exceed 0.1% (v/v).

### GFP-LC3 transfection

Podocytes were seeded into 35 mm glass bottom cell culture dish and grown to 50% confluence. Cells were transfected with Premo™ Autophagy Sensor LC3B-GFP (Invitrogen, Carlsbad, CA, USA) for 16 hours as per the manufacturer’s protocol. The transfected cells were treated under the indicated conditions and images of GFP-LC3 fluorescence were captured using a LEICA laser scan microscope (LEICA DM IRB; Leica, Wetzlar, Germany).

### Apoptosis assay by flow cytometry

Podocytes were plated into 12-well dishes and cultured under the indicated conditions. Cell apoptosis was assessed using FITC Annexin V Apoptosis Detection Kit I (Cat: 556547, BD Biosciences, Franklin Lakes, NJ, USA) following the manufacturer’s protocol. Apoptotic podocytes were defined as annexin V-positive/PI-negative (early apoptotic) and annexin V-positive/PI-positive (late apoptotic) cells.

### SERCA activity measurement

ER fraction from kidney tissue was isolated using the ER isolation kit (Cat: ER0100, Sigma Aldrich, St. Louis, MO, USA). Briefly, fresh kidney tissue was homogenized in isotonic extraction buffer in a glass tube homogenizer. After a series of centrifugation (1,000 × g for 10 min, 12,000 × g for 15 min, 100,000 × g for 1 h), the microsomes were obtained and then homogenized with isotonic extraction buffer. SERCA activity was measured based on the inorganic phosphate production using a commercially available kit (Nanjing Jiancheng Bioengineering Institute, Nanjing, China) according to the manufacturer’s instruction. SERCA activity was normalized to protein concentration.

### Statistical analysis

Data are expressed as the means ± SEM, with n representing the number of animals for *in vivo* experiments and independent experiments for *in vitro* experiments. Statistical analysis was conducted with GraphPad Prism software 5 software (GraphPad Software Inc., San Diego, CA, USA). Unpaired two-tailed t test or one-way ANOVA followed by the Newman-Keuls multiple comparisons test was used for statistical comparisons among experimental groups, with a value of *P* < 0.05 being considered statistically significant.

## Electronic supplementary material

Supporting information
