# Supplementary material for: Astragaloside IV protects against podocyte injury via SERCA2-dependent ER stress reduction and AMPKα-regulated autophagy induction in streptozotocin-induced diabetic nephropathy
Source: Sci Rep. 2017 Jul 31;7:6852. doi: 10.1038/s41598-017-07061-7 (PMC5537362; doi:10.1038/s41598-017-07061-7)
Supplement: Supplementary file 1 — Supporting information [file 41598_2017_7061_MOESM1_ESM.pdf]

# Astragaloside IV protects against podocyte injury via SERCA2-dependent ER stress reduction and AMPKα-regulated autophagy induction in streptozotocin-induced diabetic nephropathy

Hengjiang Guo[1, #], Yi Wang[2, #], Xuemei Zhang[3], Yingjun Zang[2], Yang Zhang[2], Li Wang[1], Hao Wang[2], Yunman Wang[2], Aili Cao[1,*], Wen Peng[1,2,*]

[1]Laboratory of Renal Disease, [2]Department of Nephrology, Putuo Hospital, Shanghai University of Traditional Chinese Medicine, Shanghai, China

[3]Department of Pharmacology, School of Pharmacy, Fudan University, Shanghai 201203, China

[#] These authors contributed equally to this work.

*** Correspondence:**
Wen Peng
pengwen_01@vip.sina.com
Aili Cao
Caoaili0312@sina.cn

**Supplementary Figure S1.** AS-IV alleviated renal histopathology and inflammation in STZ-induced diabetic mice.

**Supplementary Figure S2.** The original blots for cropped blots presented in Figure 1.

**Supplementary Figure S3.** The original blots for cropped blots presented in Figure 2.

**Supplementary Figure S4.** The original blots for cropped blots presented in Figure 3.

**Supplementary Figure S5.** The original blots for cropped blots presented in Figure 4.

**Supplementary Figure S6.** The original blots for cropped blots presented in Figure 5.

**Supplementary Figure S7.** The original blots for cropped blots presented in Figure 6.

**Supplementary Figure S8.** The original blots for cropped blots presented in Figure 7.

**Supplementary Figure S9.** The original blots for cropped blots presented in Figure 8.

**Supplementary Figure S10.** The original blots for cropped blots presented in Figure 9.

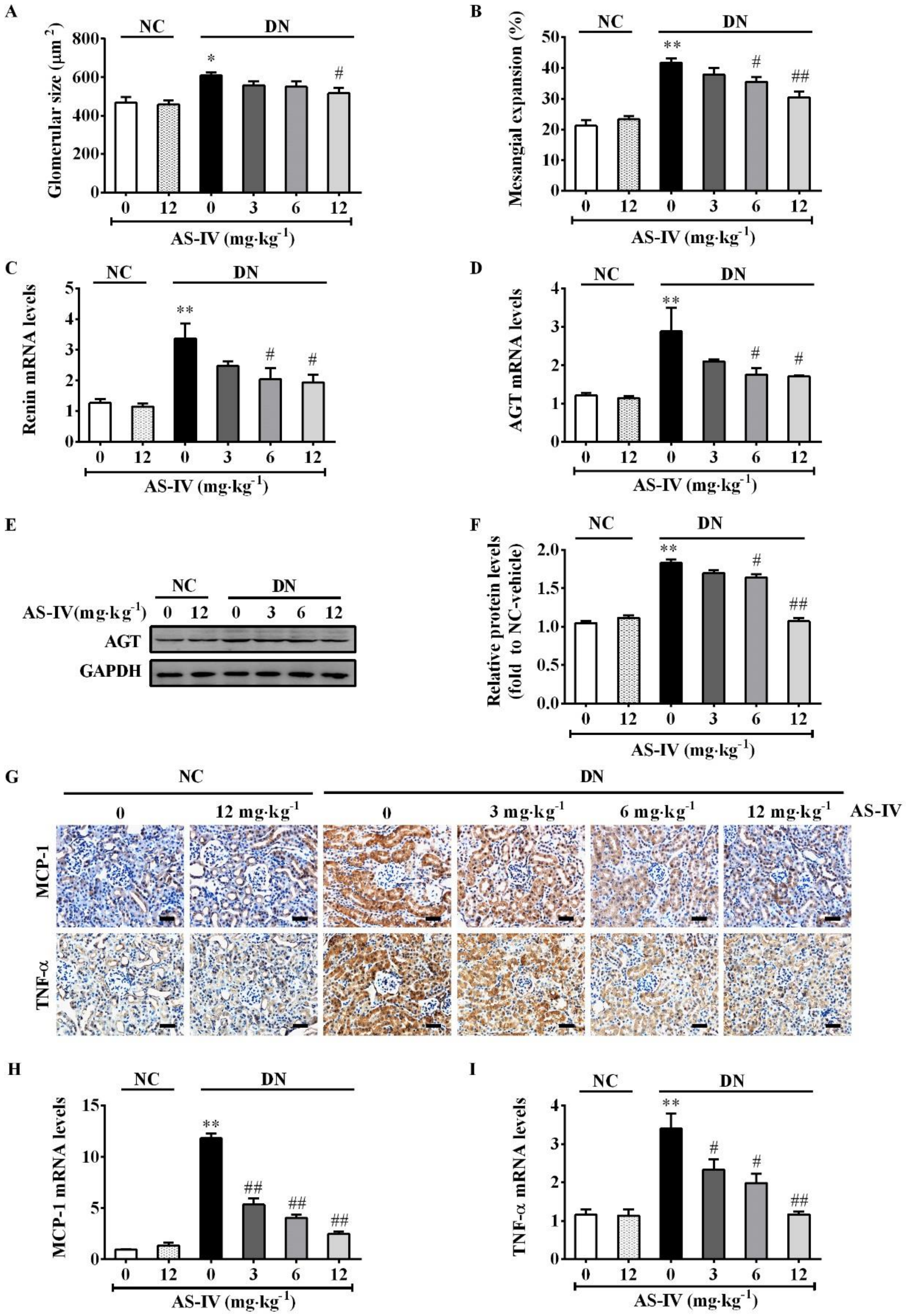


**Supplementary Figure S1. AS-IV alleviated renal histopathology and inflammation in STZ-induced diabetic mice.** (A) Glomerular size in each group. (B) Mesangial expansion in each group. Data are expressed as the percentage of the mesangial matrix area/total glomerular area. (C) The relative expression of Renin

mRNA in renal cortex. (D) The relative expression of AGT mRNA in renal cortex. (E) Western blot analyses of AGT expression in each group. (F) Densitometric quantification of AGT expression in each group. (G) Representative images of immunohistochemistry staining for MCP-1 and TNF-α. Scale bars, 20 μm; (H) The relative expression of MCP-1 mRNA in renal cortex. (I) The relative expression of TNF-α mRNA in renal cortex. n=4-6 per group. $^{*}P$ <0.05, $^{**}P < 0.01$ compared to NC-vehicle group; $^{\#}P < 0.05$, $^{\#\#}P < 0.01$ compared to DN-vehicle group. One-way ANOVA and Newman-Keuls multiple comparisons test (A, B, D, E). NC, nondiabetic control. DN, diabetic nephropathy.

A

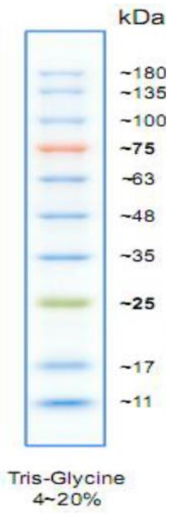


B

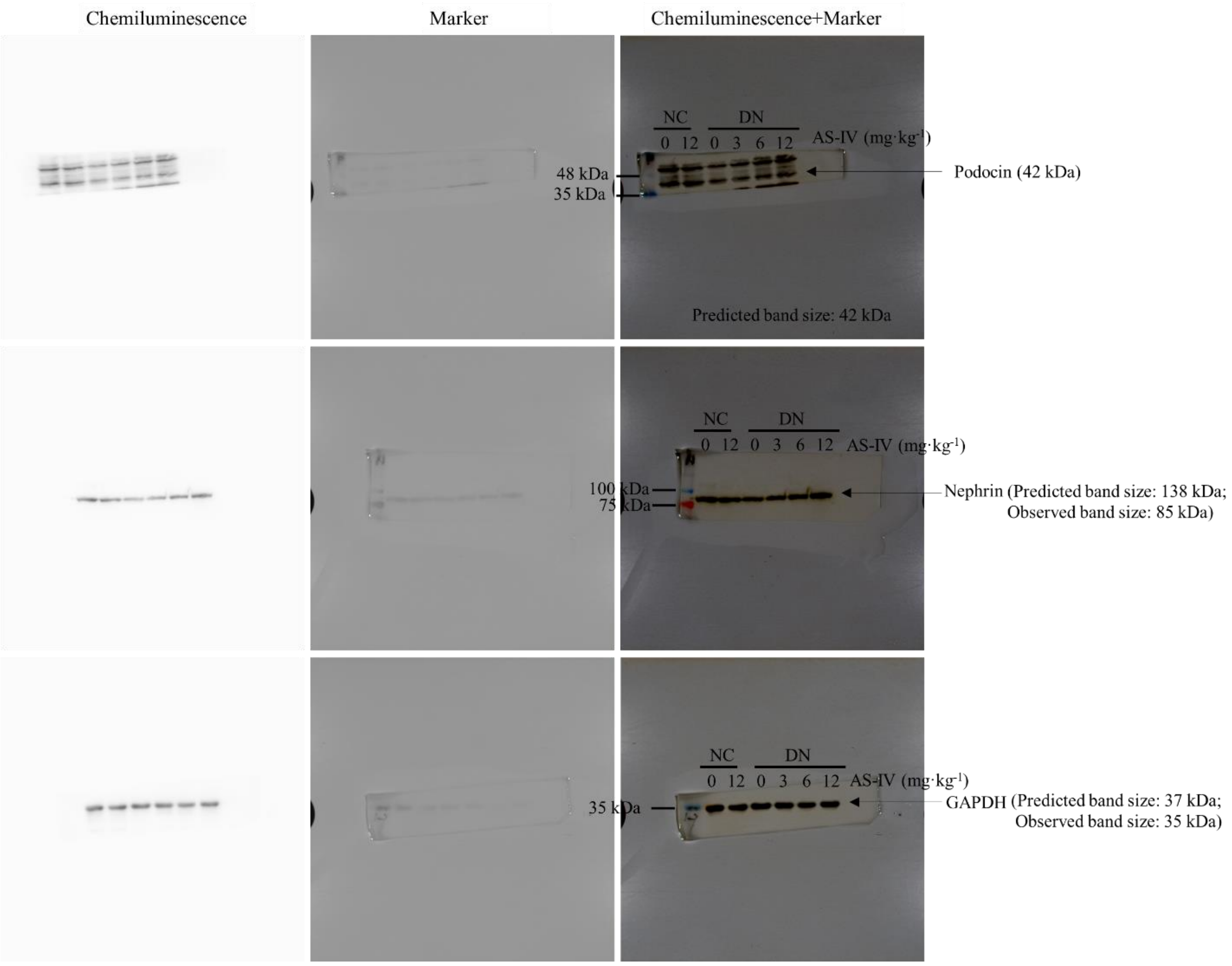


**Supplementary Figure S2. The original blots for cropped blots presented in Figure 1.** (A) Prestained dual color protein molecular weight marker. (B) The original blots for panel I presented in Figure 1 in the main text. NC, nondiabetic control. DN, diabetic nephropathy.

**A**

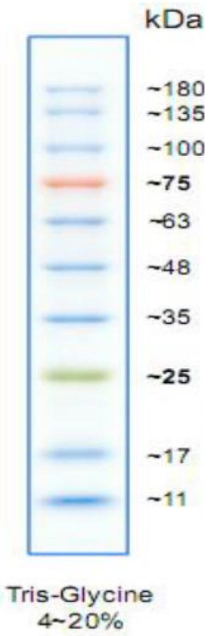
kDa
~180
~135
~100
~75
~63
~48
~35
~25
~17
~11
Tris-Glycine
4~20%

**B**

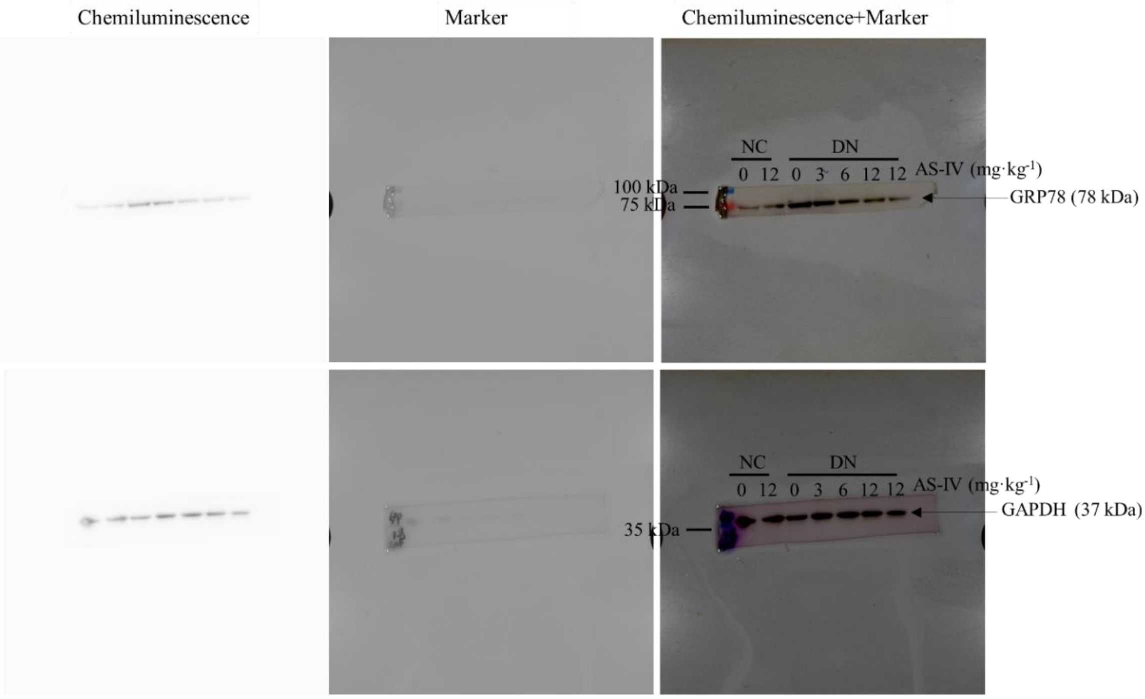
Chemiluminescence
Marker
Chemiluminescence+Marker
NC
DN
0 12 0 3 6 12 12 AS-IV (mg·kg-1)
100 kDa
75 kDa
GRP78 (78 kDa)
NC
DN
0 12 0 3 6 12 12 AS-IV (mg·kg-1)
35 kDa
GAPDH (37 kDa)

C

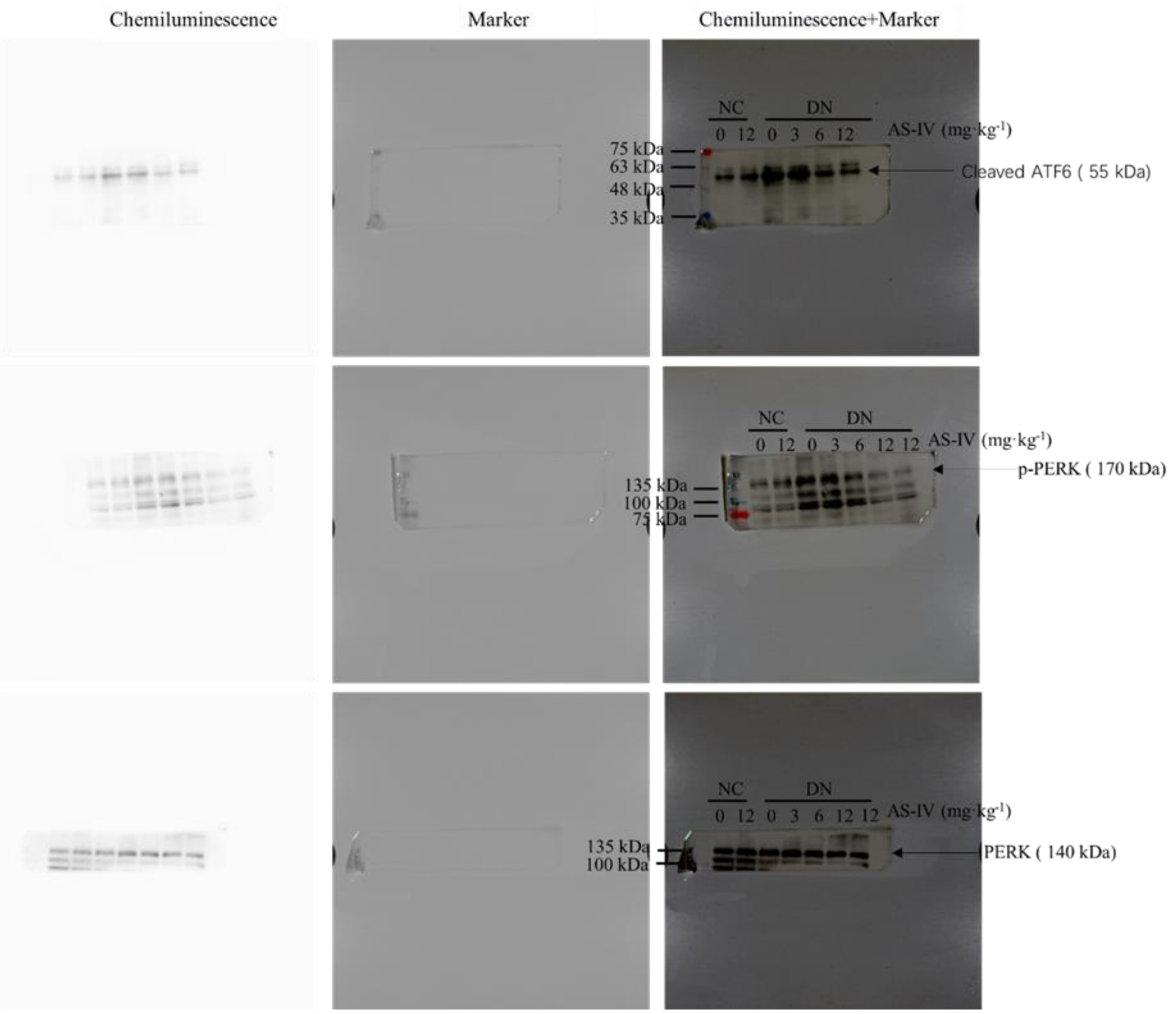
Chemiluminescence
Marker
Chemiluminescence+Marker
NC
DN
0 12 0 3 6 12
AS-IV (mg·kg-1)
75 kDa
63 kDa
48 kDa
35 kDa
Cleaved ATF6 ( 55 kDa)
NC
DN
0 12 0 3 6 12 12
AS-IV (mg·kg-1)
135 kDa
100 kDa
75 kDa
p-PERK ( 170 kDa)
NC
DN
0 12 0 3 6 12 12
AS-IV (mg·kg-1)
135 kDa
100 kDa
PERK ( 140 kDa)

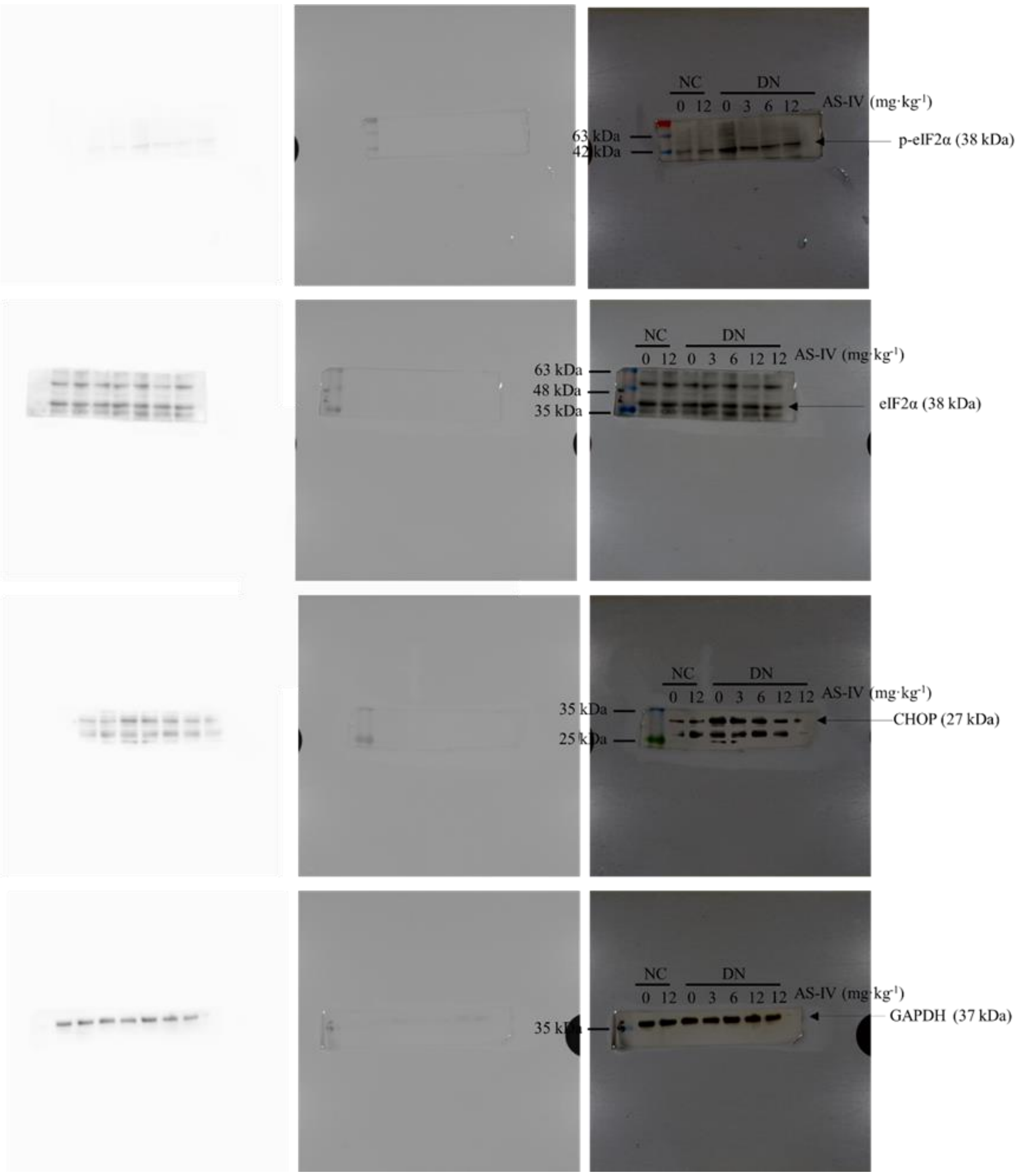
NC
DN
0 12 0 3 6 12
AS-IV (mg·kg-1)
63 kDa
42 kDa
p-eIF2α (38 kDa)
NC
DN
0 12 0 3 6 12 12
AS-IV (mg·kg-1)
63 kDa
48 kDa
35 kDa
eIF2α (38 kDa)
NC
DN
0 12 0 3 6 12 12
AS-IV (mg·kg-1)
35 kDa
25 kDa
CHOP (27 kDa)
NC
DN
0 12 0 3 6 12 12
AS-IV (mg·kg-1)
35 kDa
GAPDH (37 kDa)

**D**

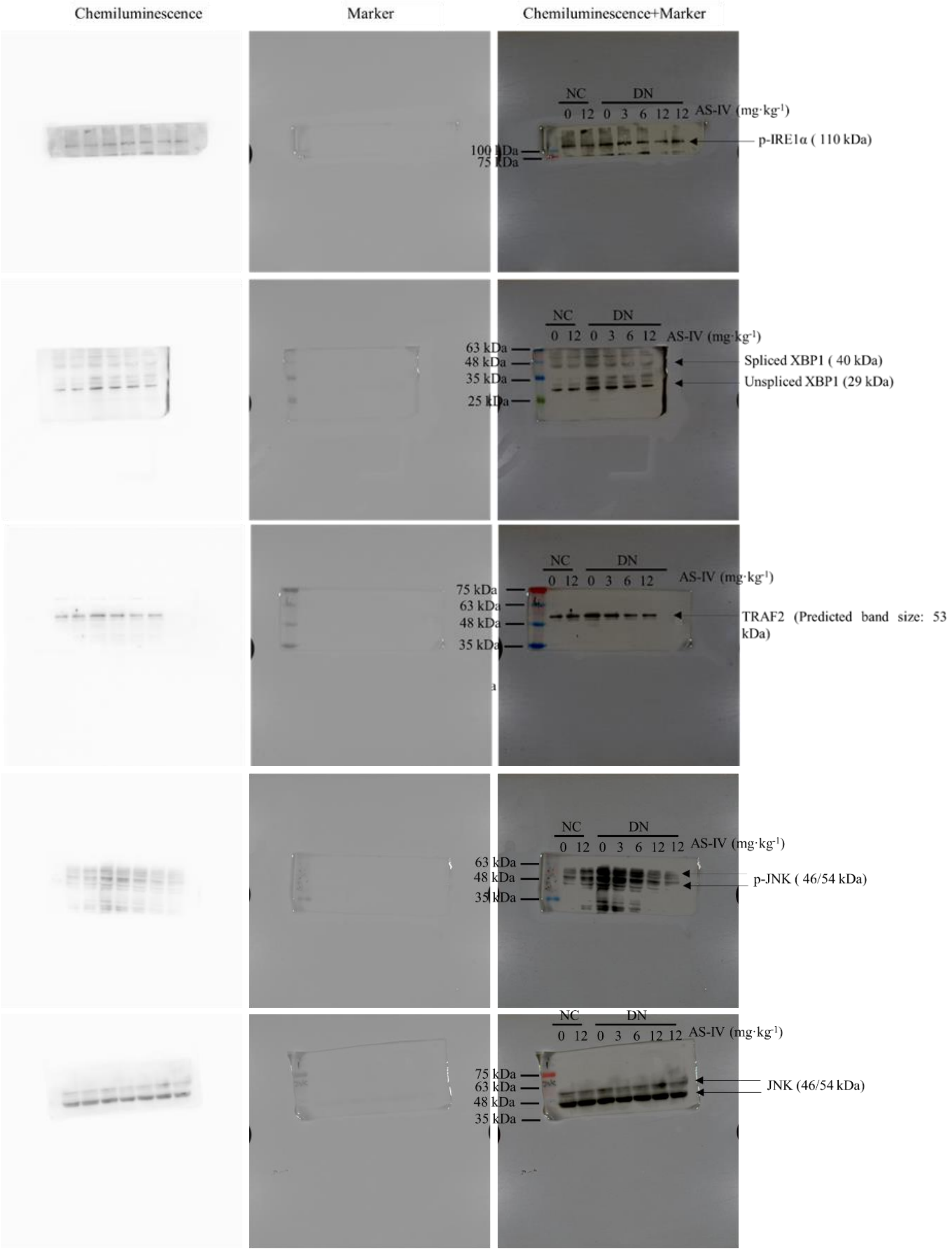

Chemiluminescence
Marker
Chemiluminescence+Marker
NC
DN
0 12 0 3 6 12 12 AS-IV (mg·kg-1)
p-IRE1α ( 110 kDa)
100 kDa
75 kDa
NC
DN
0 12 0 3 6 12 AS-IV (mg·kg-1)
63 kDa
48 kDa
35 kDa
25 kDa
Spliced XBP1 ( 40 kDa)
Unspliced XBP1 (29 kDa)
NC
DN
0 12 0 3 6 12 AS-IV (mg·kg-1)
75 kDa
63 kDa
48 kDa
35 kDa
TRAF2 (Predicted band size: 53 kDa)
NC
DN
0 12 0 3 6 12 12 AS-IV (mg·kg-1)
63 kDa
48 kDa
35 kDa
p-JNK ( 46/54 kDa)
NC
DN
0 12 0 3 6 12 12 AS-IV (mg·kg-1)
75 kDa
63 kDa
48 kDa
35 kDa
JNK (46/54 kDa)

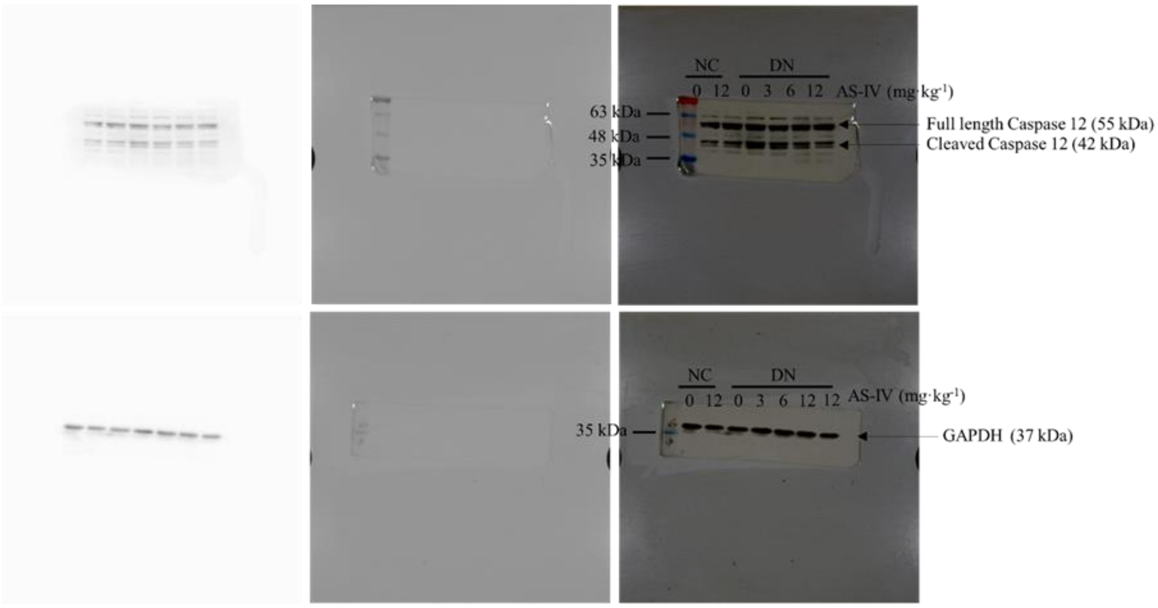


**Supplementary Figure S3. The original blots for cropped blots presented in Figure 2.** (A) Prestained dual color protein molecular weight marker. (B) The original blots for panel B presented in Figure 2 in the main text. (C) The original blots for panel D presented in Figure 2 in the main text. (D) The original blots for panel F presented in Figure 2 in the main text. NC, nondiabetic control. DN, diabetic nephropathy.

**A**

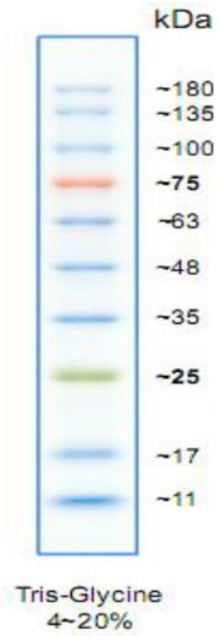


**B**

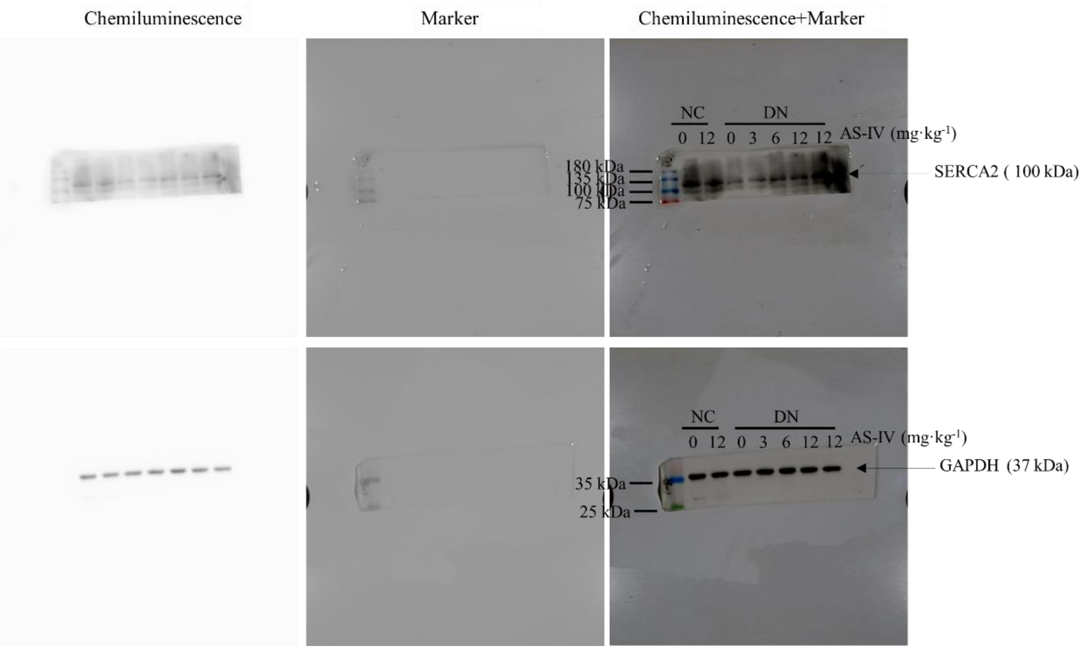


**Supplementary Figure S4. The original blots for cropped blots presented in Figure 3.** (A) Prestained dual color protein molecular weight marker. (B) The original blots for panel E presented in Figure 3 in the main text. NC, nondiabetic control. DN, diabetic nephropathy.

A

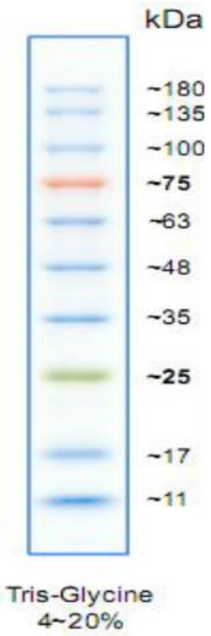

**B**

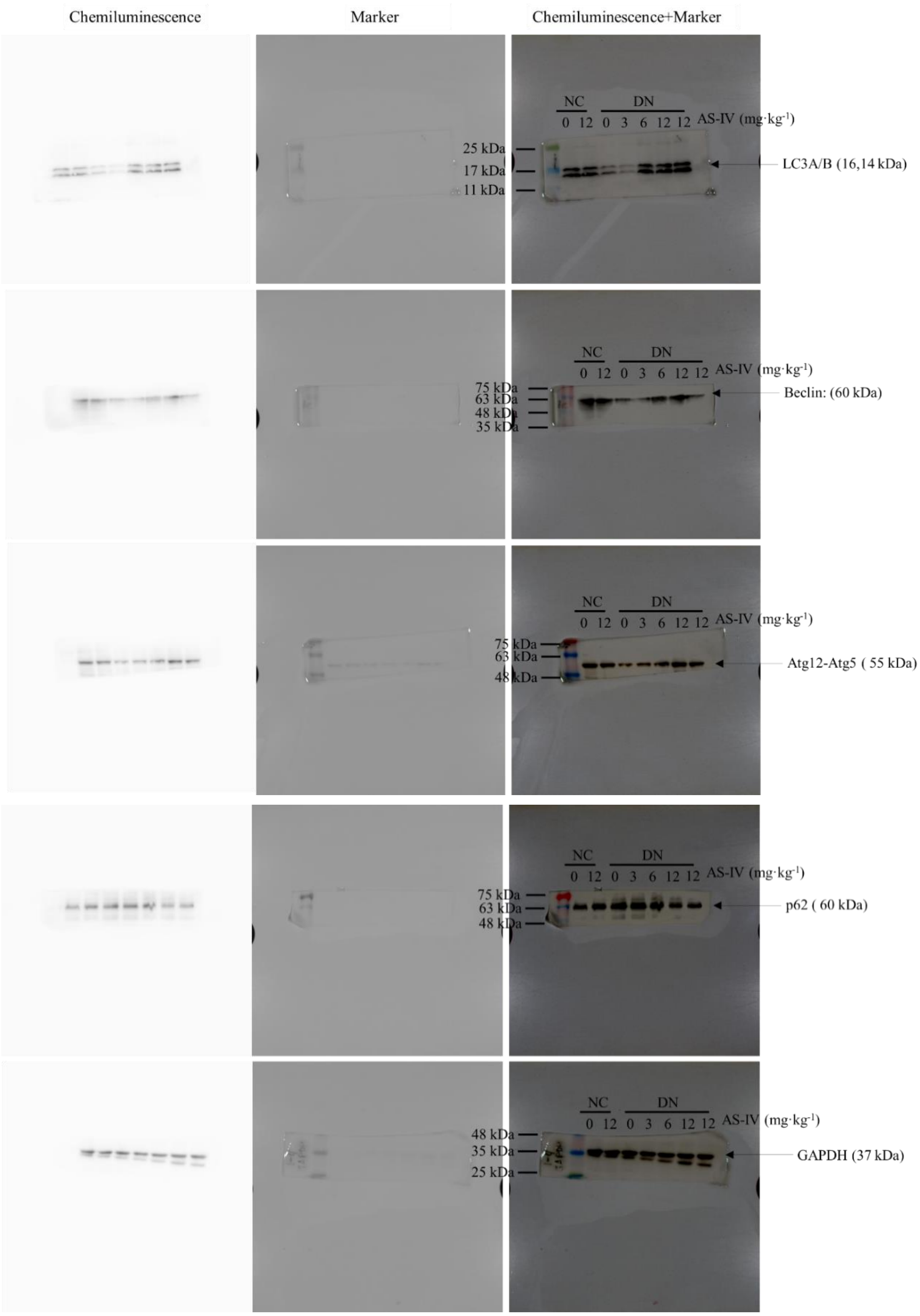

Chemiluminescence
Marker
Chemiluminescence+Marker
NC
DN
0 12 0 3 6 12 12 AS-IV (mg·kg-1)
25 kDa
17 kDa
11 kDa
LC3A/B (16,14 kDa)
75 kDa
63 kDa
48 kDa
35 kDa
Beclin: (60 kDa)
Atg12-Atg5 ( 55 kDa)
p62 ( 60 kDa)
GAPDH (37 kDa)

C.

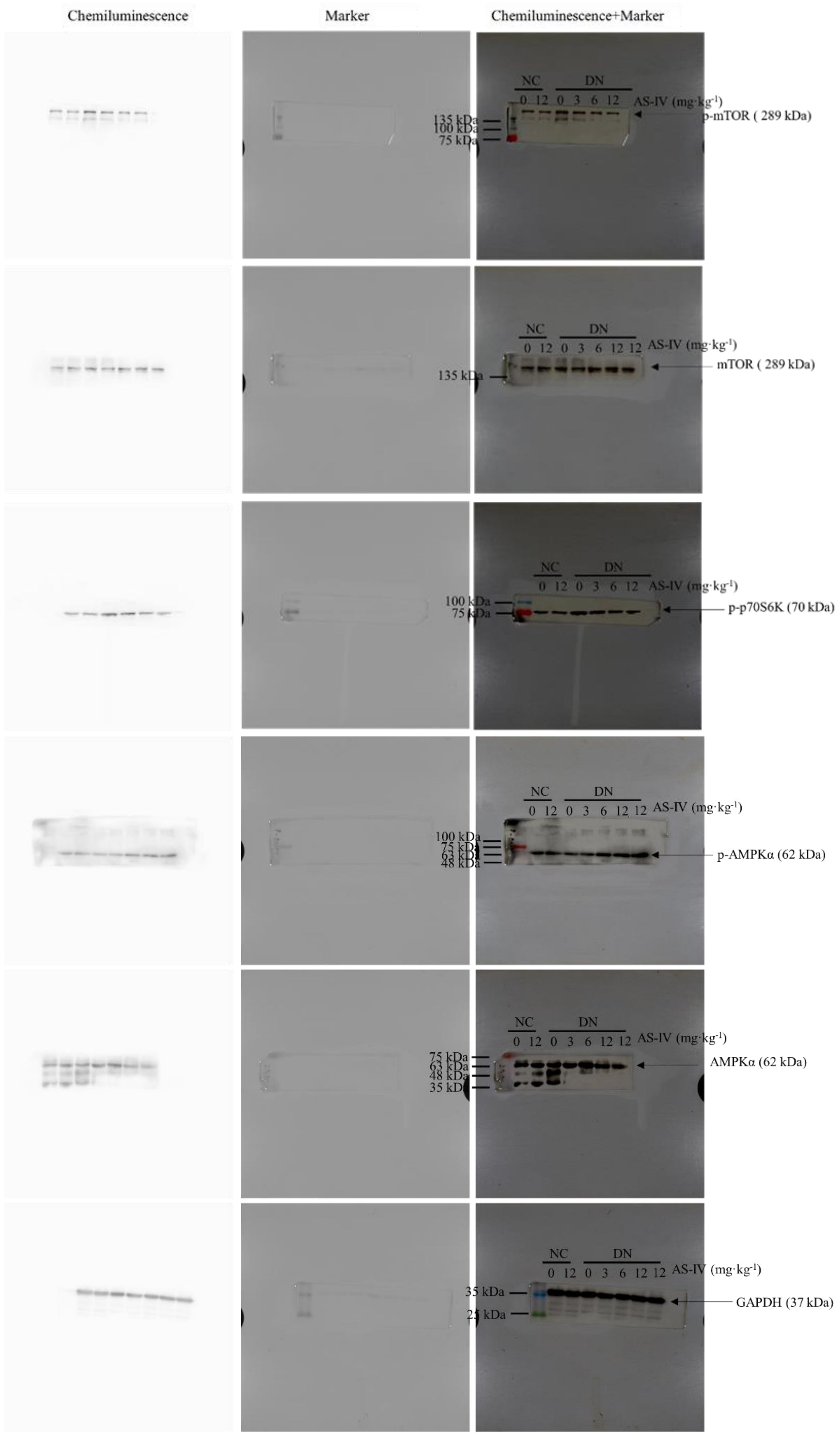
Chemiluminescence
Marker
Chemiluminescence+Marker
NC
DN
0 12 0 3 6 12
AS-IV (mg·kg-1)
135 kDa
100 kDa
75 kDa
p-mTOR ( 289 kDa)
0 12 0 3 6 12 12
135 kDa
mTOR ( 289 kDa)
100 kDa
75 kDa
p-p70S6K (70 kDa)
100 kDa
75 kDa
63 kDa
48 kDa
p-AMPKα (62 kDa)
75 kDa
63 kDa
48 kDa
35 kDa
AMPKα (62 kDa)
35 kDa
25 kDa
GAPDH (37 kDa)

**Supplementary Figure S5. The original blots for cropped blots presented in Figure 4.** (A) Prestained dual color protein molecular weight marker. (B) The original blots for panel C presented in Figure 4 in the main text. (C) The original blots for panel E presented in Figure 4 in the main text. NC, nondiabetic control. DN, diabetic nephropathy.

A

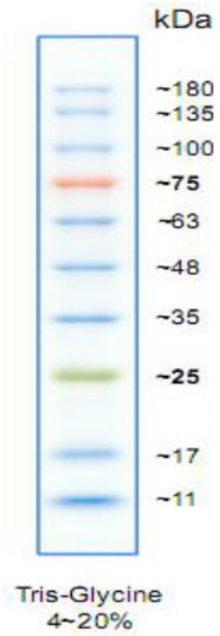
kDa
~180
~135
~100
~75
~63
~48
~35
~25
~17
~11
Tris-Glycine
4~20%

B

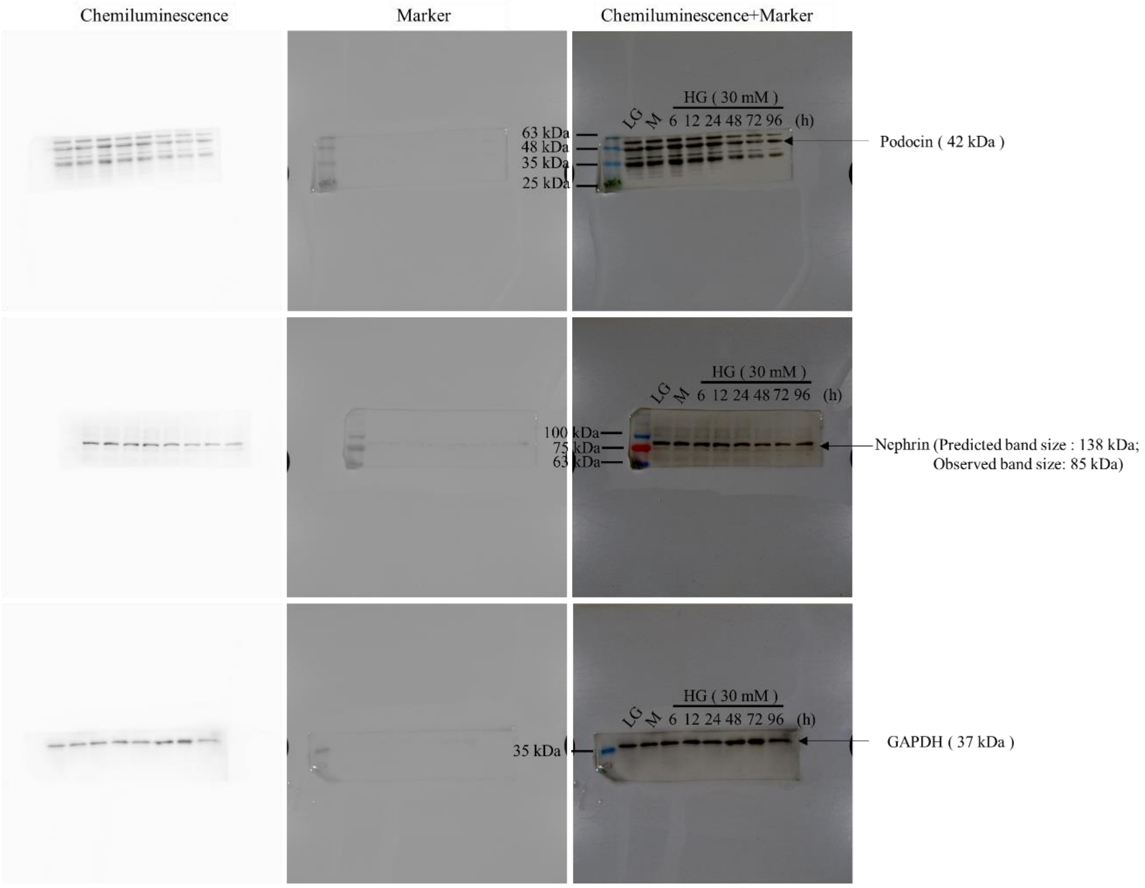
Chemiluminescence
Marker
Chemiluminescence+Marker
HG ( 30 mM )
LG M 6 12 24 48 72 96 (h)
63 kDa
48 kDa
35 kDa
25 kDa
Podocin ( 42 kDa )
100 kDa
75 kDa
63 kDa
Nephrin (Predicted band size : 138 kDa;
Observed band size: 85 kDa)
35 kDa
GAPDH ( 37 kDa )

**C**

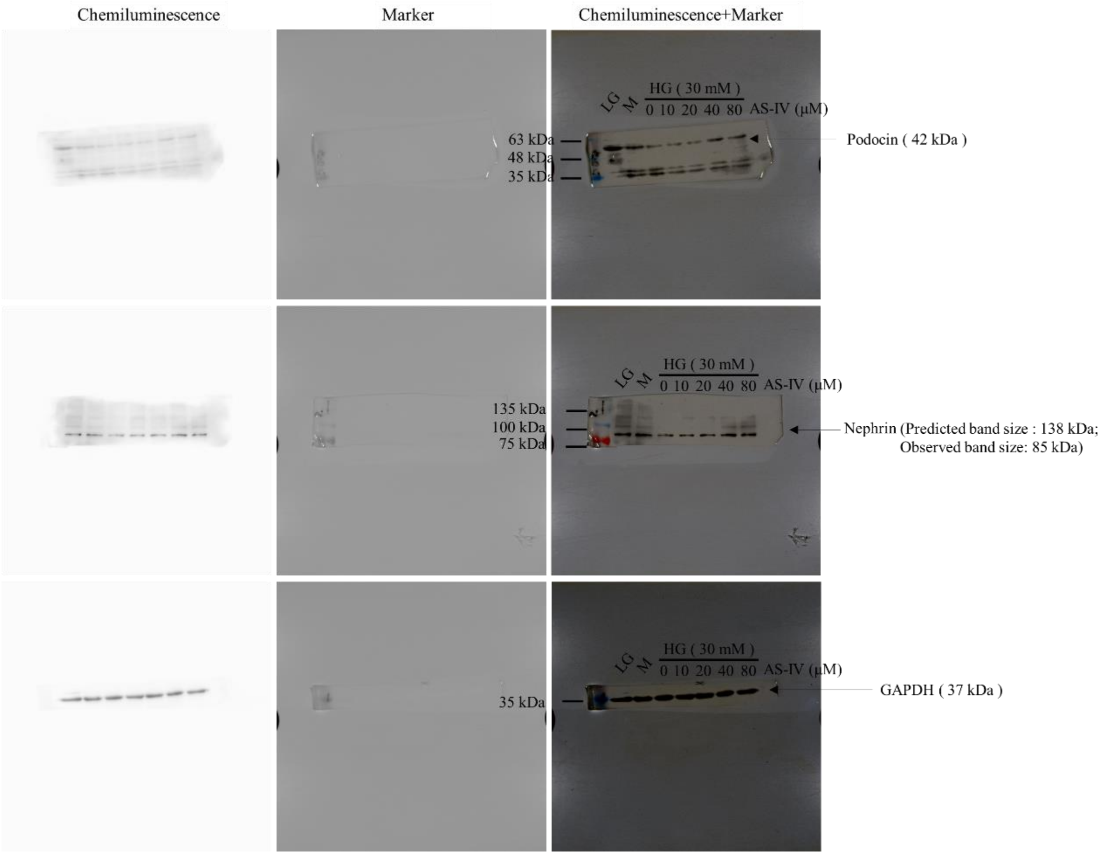


**Supplementary Figure S6. The original blots for cropped blots presented in Figure 5.** (A) Prestained dual color protein molecular weight marker. (B) The original blots for panel C presented in Figure 5 in the main text. (C) The original blots for panel G presented in Figure 5 in the main text. LG, low glucose; M, mannitol; HG, high glucose.

A

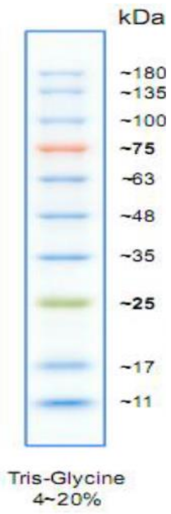

**B**

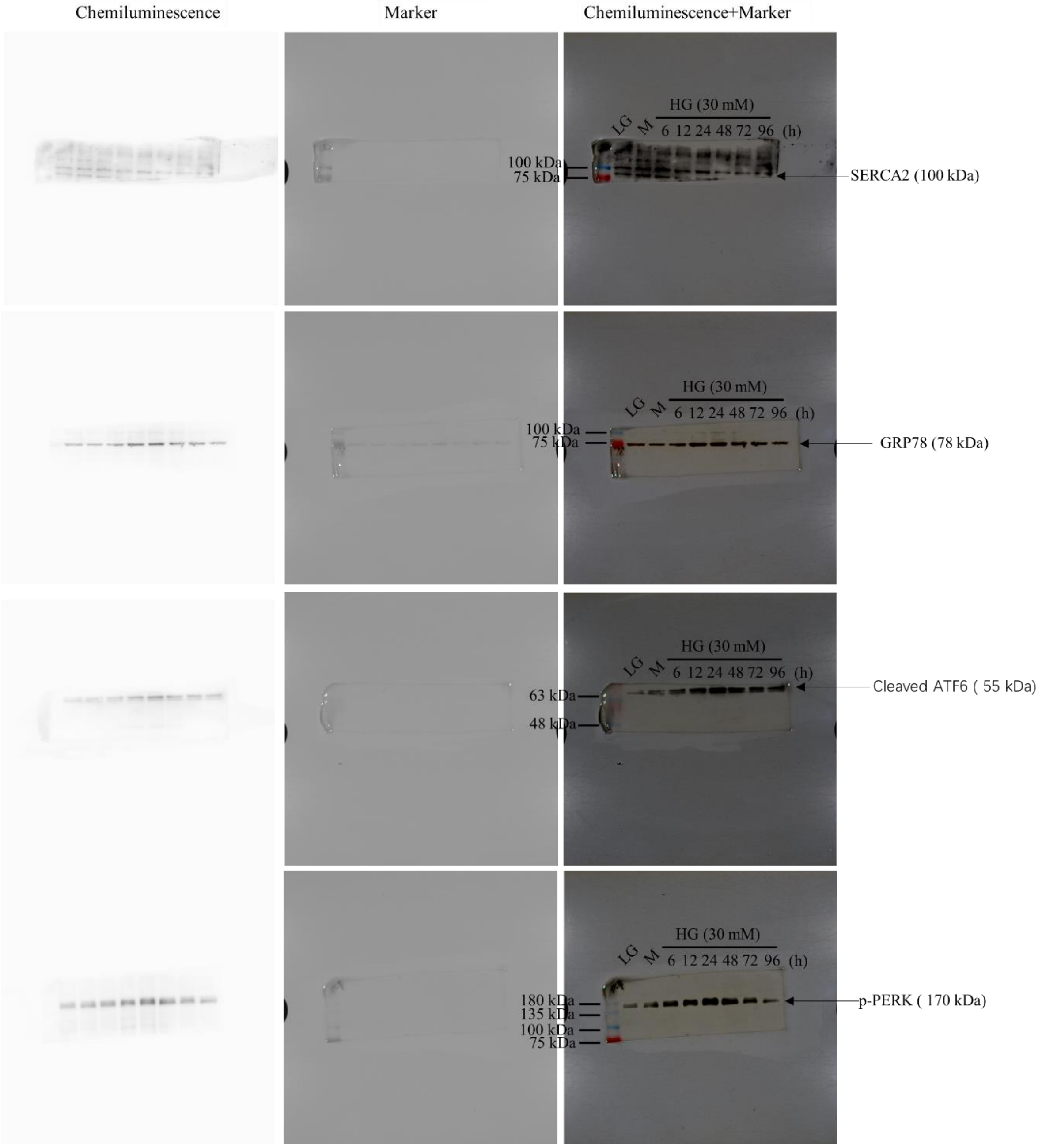
Chemiluminescence
Marker
Chemiluminescence+Marker
HG (30 mM)
LG M 6 12 24 48 72 96 (h)
100 kDa
75 kDa
SERCA2 (100 kDa)
HG (30 mM)
LG M 6 12 24 48 72 96 (h)
100 kDa
75 kDa
GRP78 (78 kDa)
HG (30 mM)
LG M 6 12 24 48 72 96 (h)
63 kDa
48 kDa
Cleaved ATF6 ( 55 kDa)
HG (30 mM)
LG M 6 12 24 48 72 96 (h)
180 kDa
135 kDa
100 kDa
75 kDa
p-PERK ( 170 kDa)

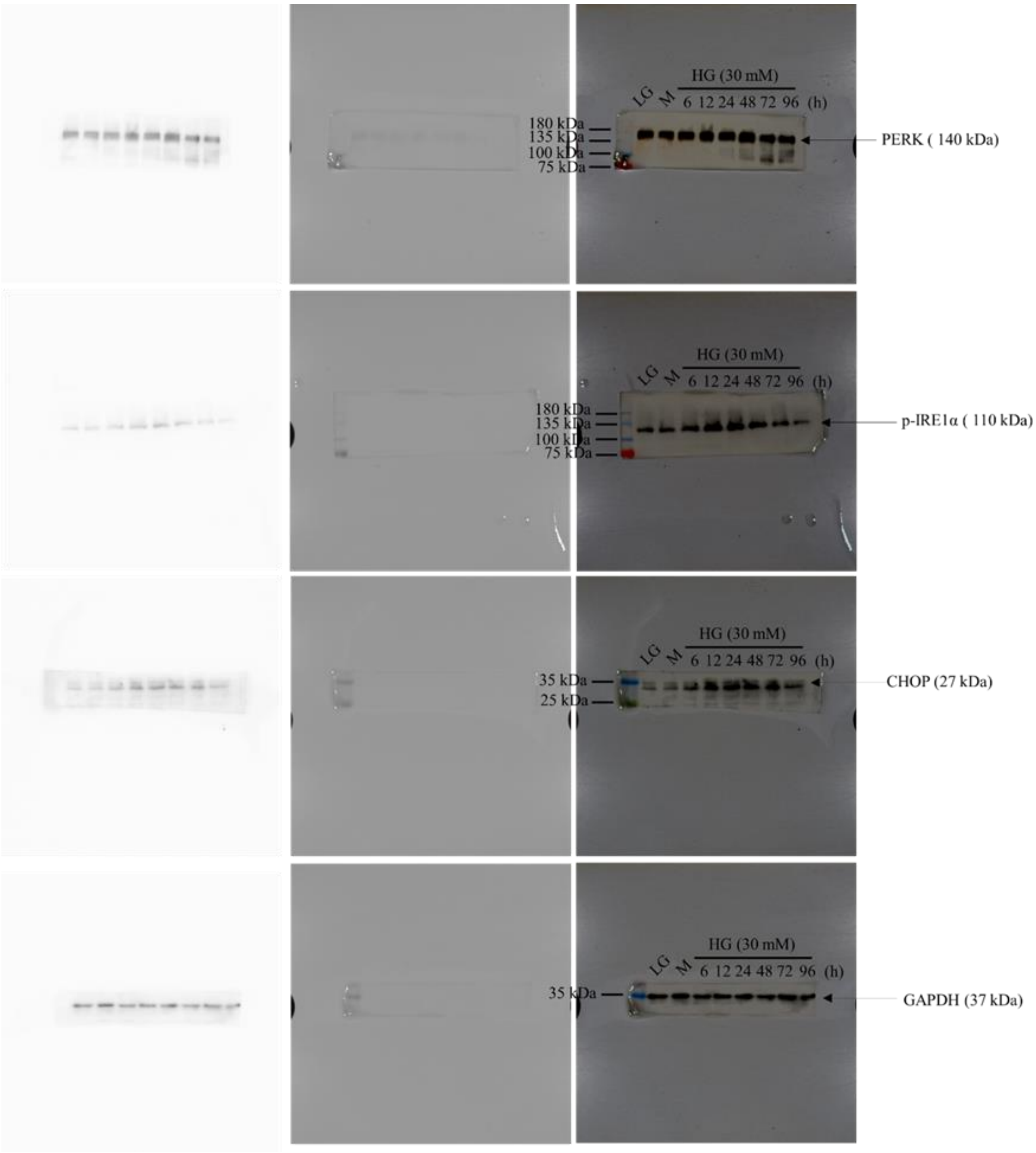
HG (30 mM)
LG M 6 12 24 48 72 96 (h)
180 kDa
135 kDa
100 kDa
75 kDa
PERK ( 140 kDa)
HG (30 mM)
LG M 6 12 24 48 72 96 (h)
180 kDa
135 kDa
100 kDa
75 kDa
p-IRE1α ( 110 kDa)
HG (30 mM)
LG M 6 12 24 48 72 96 (h)
35 kDa
25 kDa
CHOP (27 kDa)
HG (30 mM)
LG M 6 12 24 48 72 96 (h)
35 kDa
GAPDH (37 kDa)

**C**

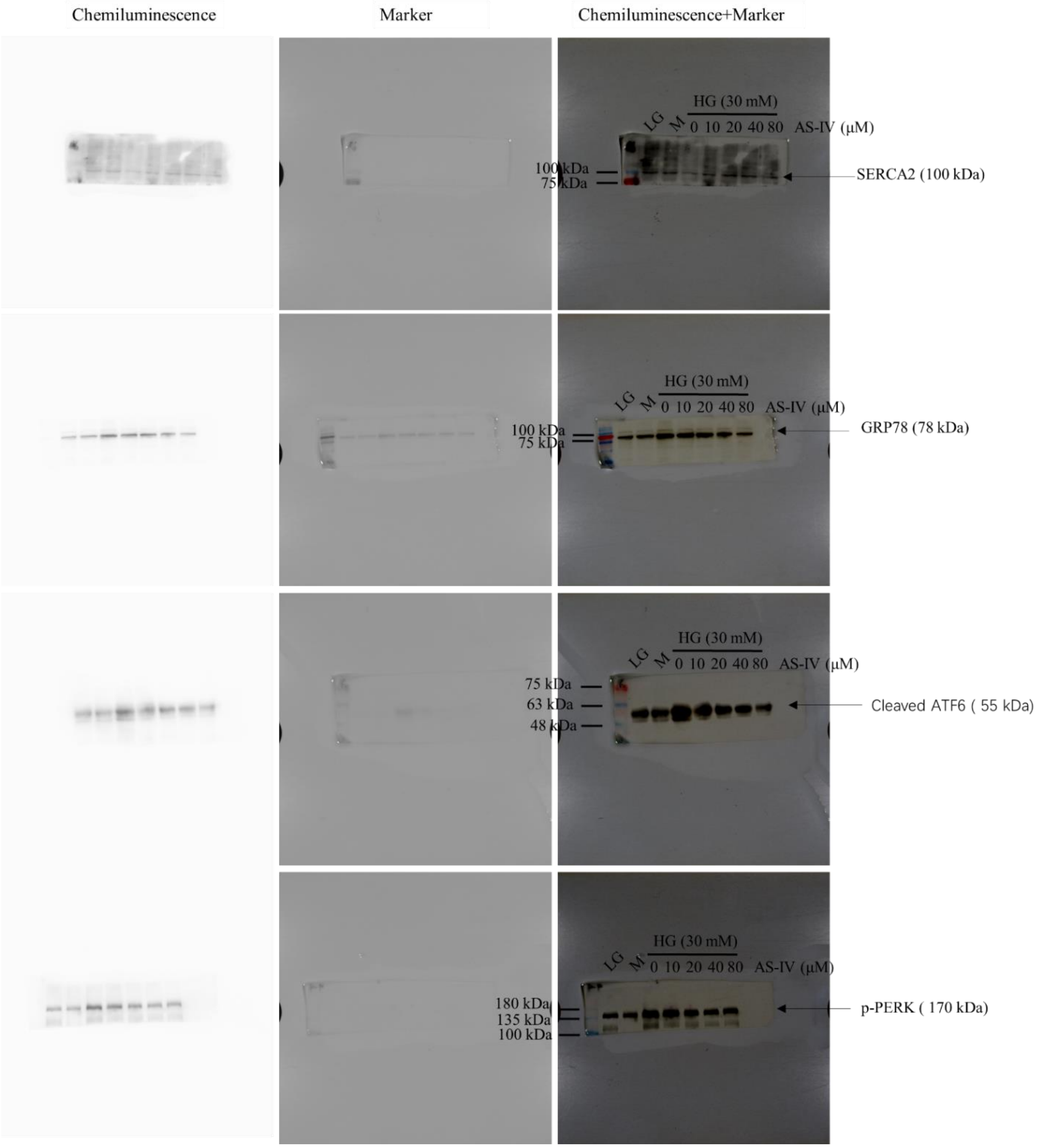

Chemiluminescence
Marker
Chemiluminescence+Marker
HG (30 mM)
LG
M
0 10 20 40 80
AS-IV (μM)
100 kDa
75 kDa
SERCA2 (100 kDa)
GRP78 (78 kDa)
63 kDa
48 kDa
Cleaved ATF6 ( 55 kDa)
180 kDa
135 kDa
p-PERK ( 170 kDa)

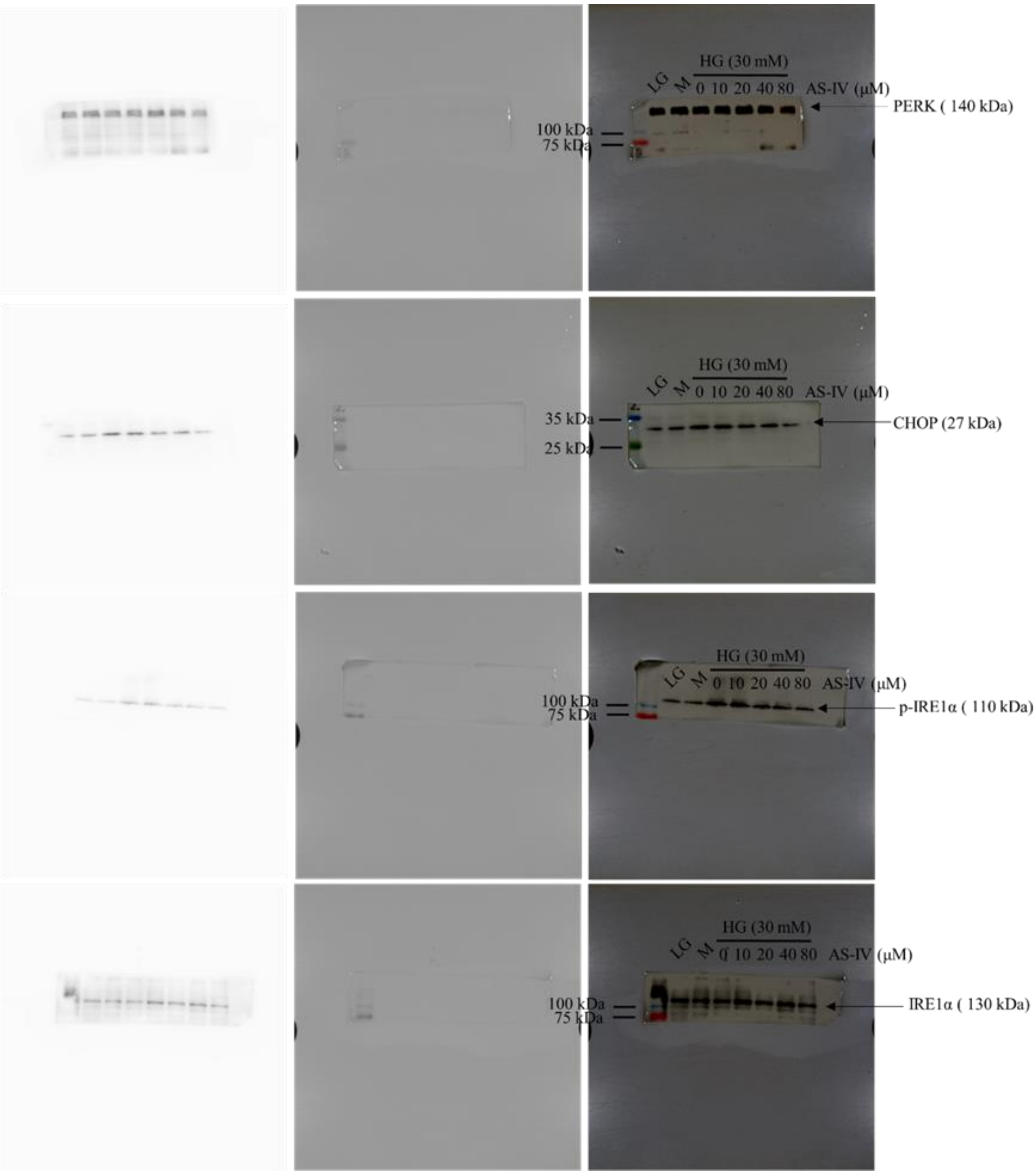
HG (30 mM)
LG
M
0 10 20 40 80
AS-IV (μM)
PERK ( 140 kDa)
100 kDa
75 kDa
HG (30 mM)
LG
M
0 10 20 40 80
AS-IV (μM)
CHOP (27 kDa)
35 kDa
25 kDa
HG (30 mM)
LG
M
0 10 20 40 80
AS-IV (μM)
p-IRE1α ( 110 kDa)
100 kDa
75 kDa
HG (30 mM)
LG
M
0 10 20 40 80
AS-IV (μM)
IRE1α ( 130 kDa)
100 kDa
75 kDa

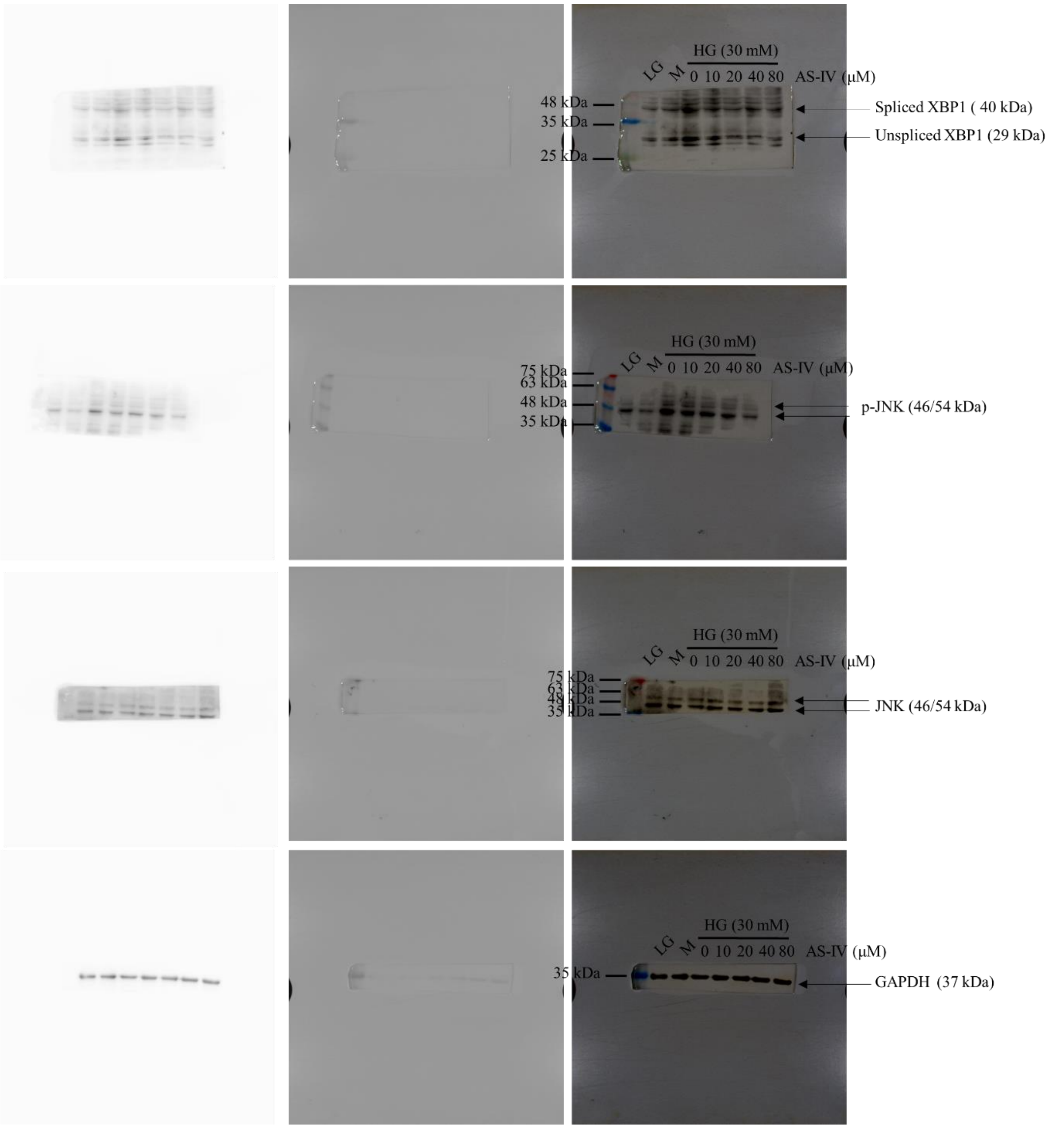

HG (30 mM)
LG M 0 10 20 40 80 AS-IV (μM)
48 kDa
35 kDa
25 kDa
Spliced XBP1 ( 40 kDa)
Unspliced XBP1 (29 kDa)
HG (30 mM)
LG M 0 10 20 40 80 AS-IV (μM)
75 kDa
63 kDa
48 kDa
35 kDa
p-JNK (46/54 kDa)
HG (30 mM)
LG M 0 10 20 40 80 AS-IV (μM)
75 kDa
63 kDa
48 kDa
35 kDa
JNK (46/54 kDa)
HG (30 mM)
LG M 0 10 20 40 80 AS-IV (μM)
35 kDa
GAPDH (37 kDa)

**D**

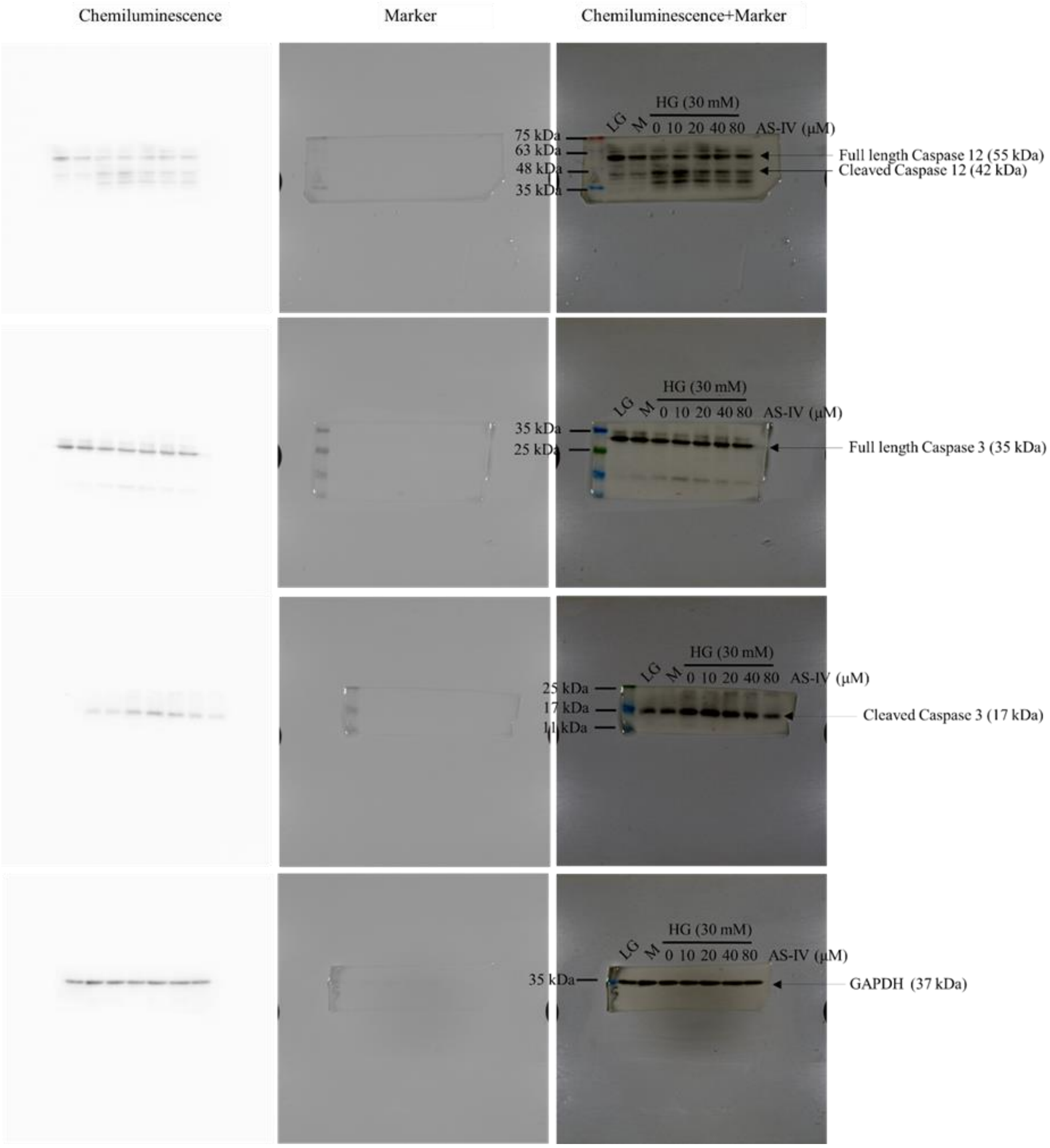


**Supplementary Figure S7. The original blots for cropped blots presented in Figure 6.** (A) Prestained dual color protein molecular weight marker. (B) The original blots for panel A presented in Figure 6 in the main text. (C) The original blots for panel C presented in Figure 6 in the main text. (D) The original blots for panel F presented in Figure 6 in the main text. LG, low glucose; M, mannitol; HG, high glucose.

A

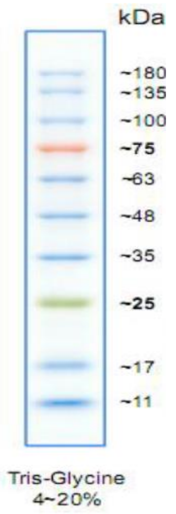
kDa
~180
~135
~100
~75
~63
~48
~35
~25
~17
~11
Tris-Glycine
4~20%

B

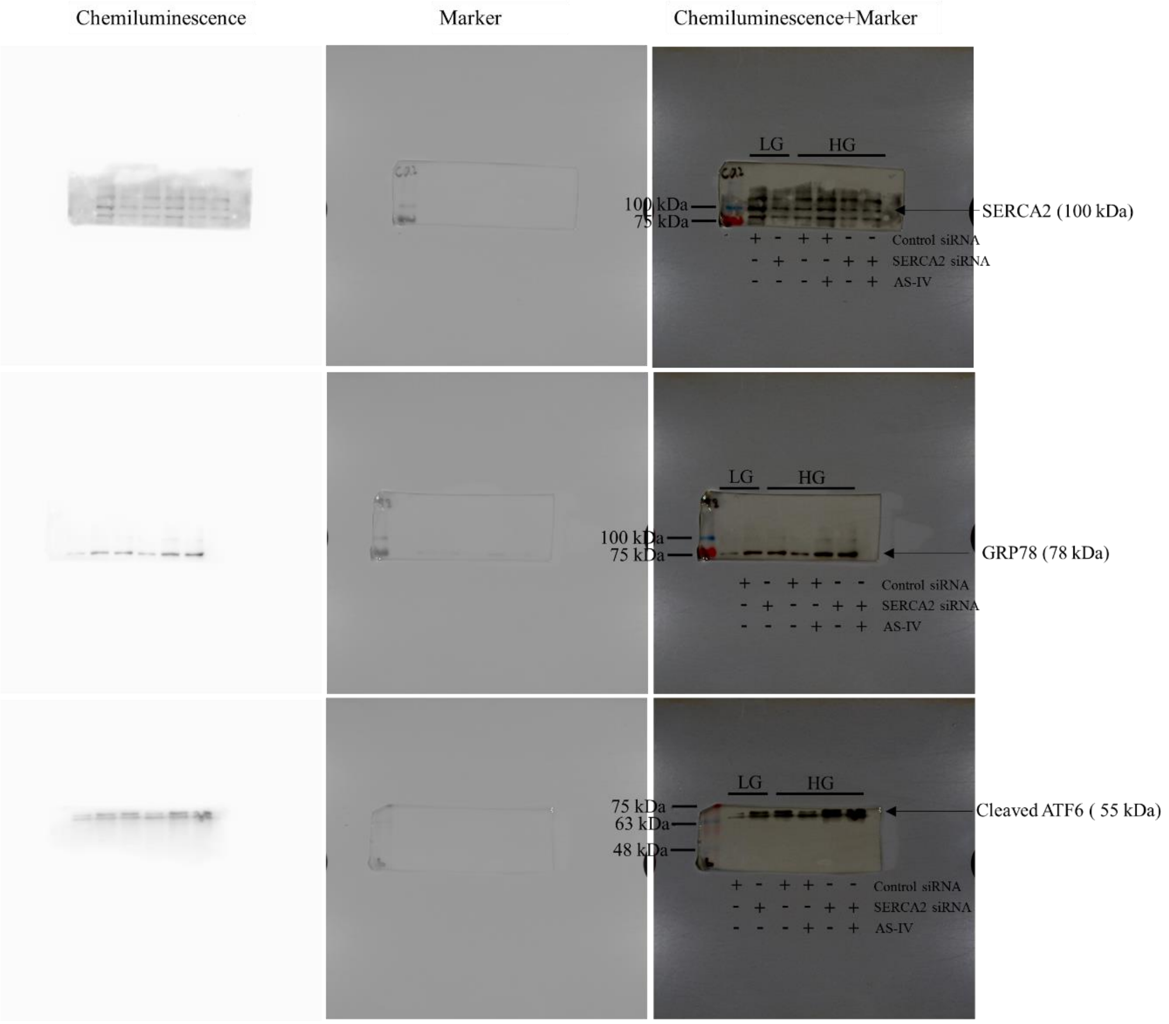
Chemiluminescence
Marker
Chemiluminescence+Marker
LG
HG
100 kDa
75 kDa
SERCA2 (100 kDa)
Control siRNA
SERCA2 siRNA
AS-IV
GRP78 (78 kDa)
63 kDa
48 kDa
Cleaved ATF6 ( 55 kDa)

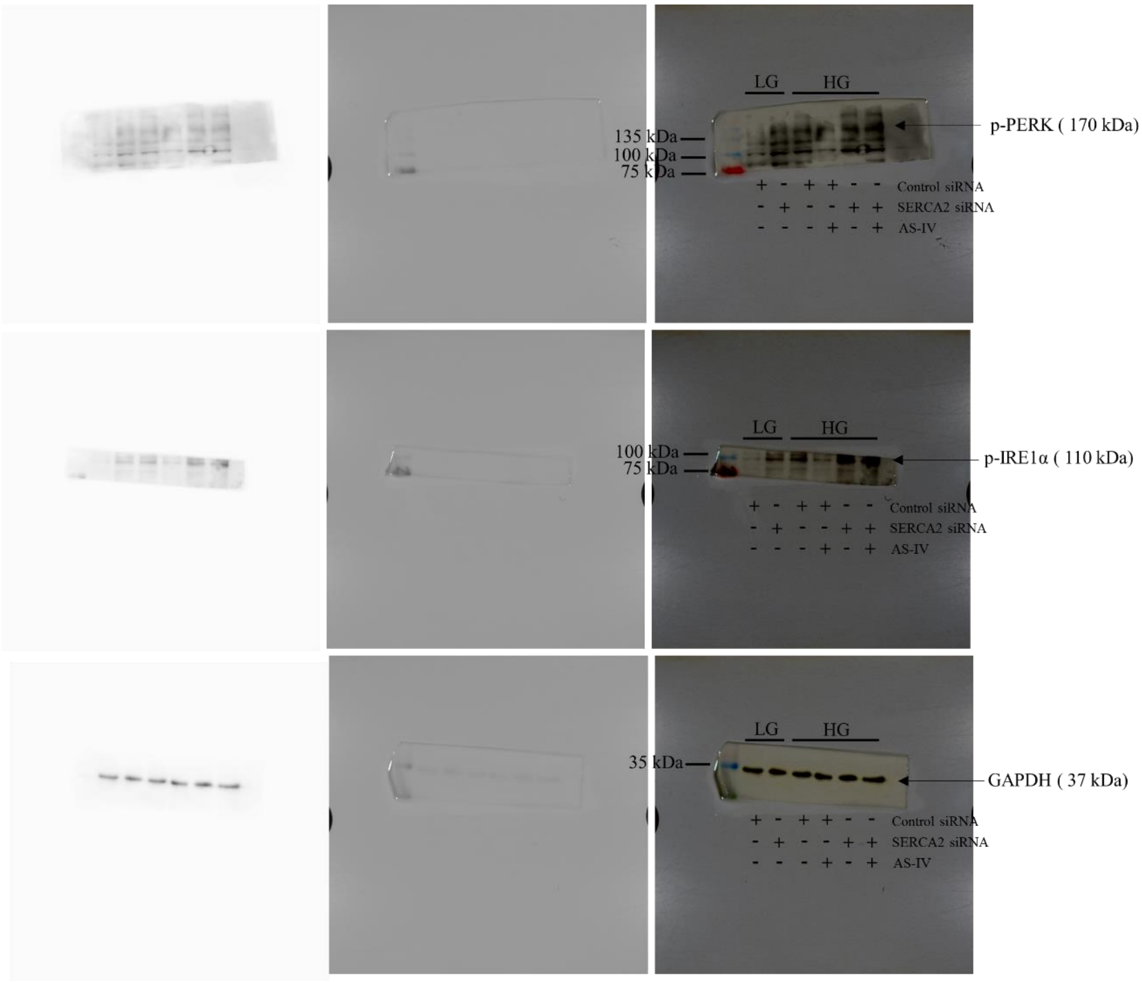


**Supplementary Figure S8. The original blots for cropped blots presented in Figure 7.** (A) Prestained dual color protein molecular weight marker. (B) The original blots for panel A presented in Figure 7 in the main text. LG, low glucose; HG, high glucose;

**A**

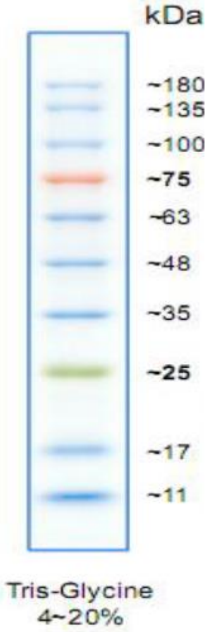

B

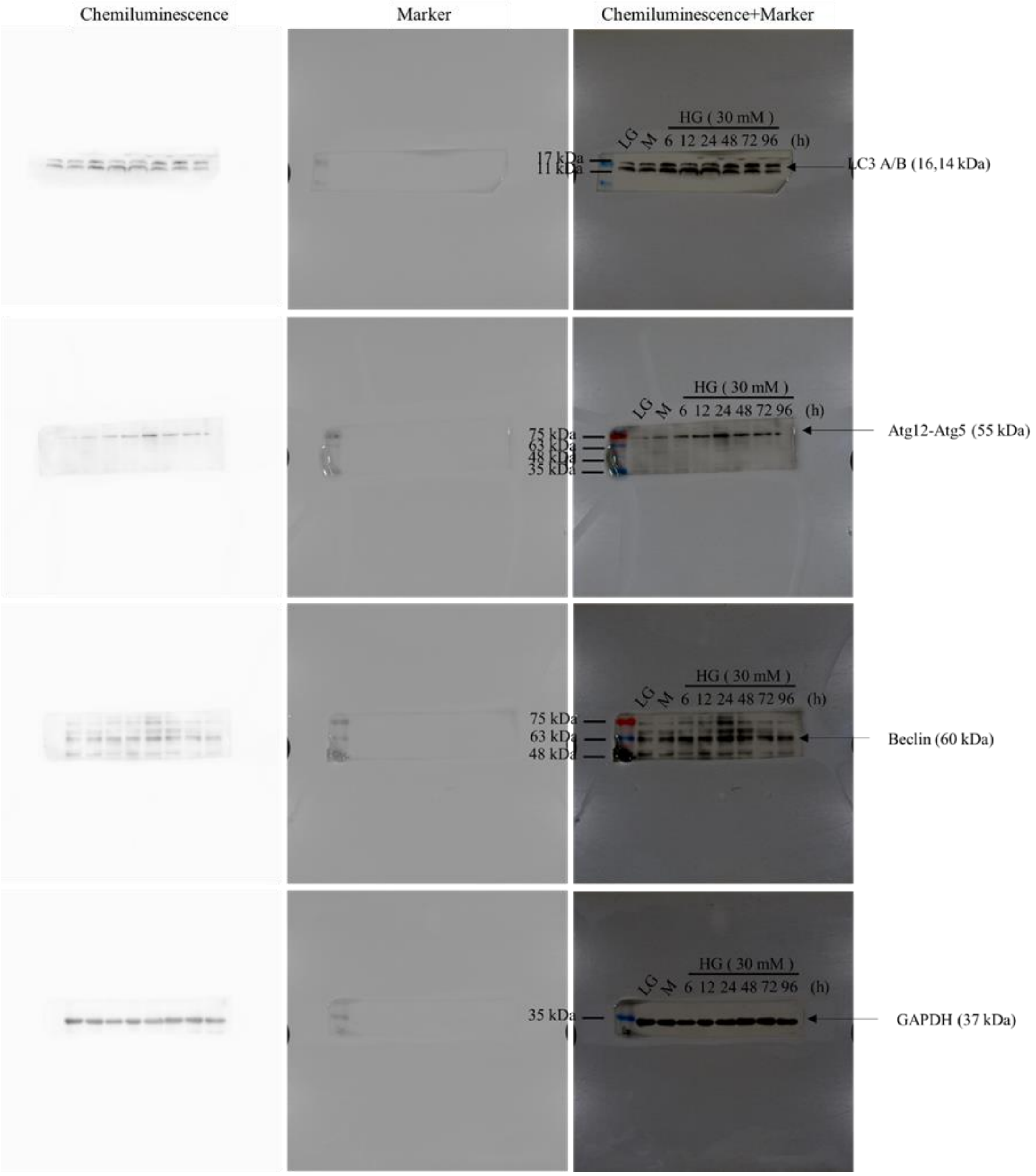
Chemiluminescence
Marker
Chemiluminescence+Marker
HG ( 30 mM )
LG M 6 12 24 48 72 96 (h)
17 kDa
11 kDa
LC3 A/B (16,14 kDa)
75 kDa
63 kDa
48 kDa
35 kDa
Atg12-Atg5 (55 kDa)
75 kDa
63 kDa
48 kDa
Beclin (60 kDa)
35 kDa
GAPDH (37 kDa)

C

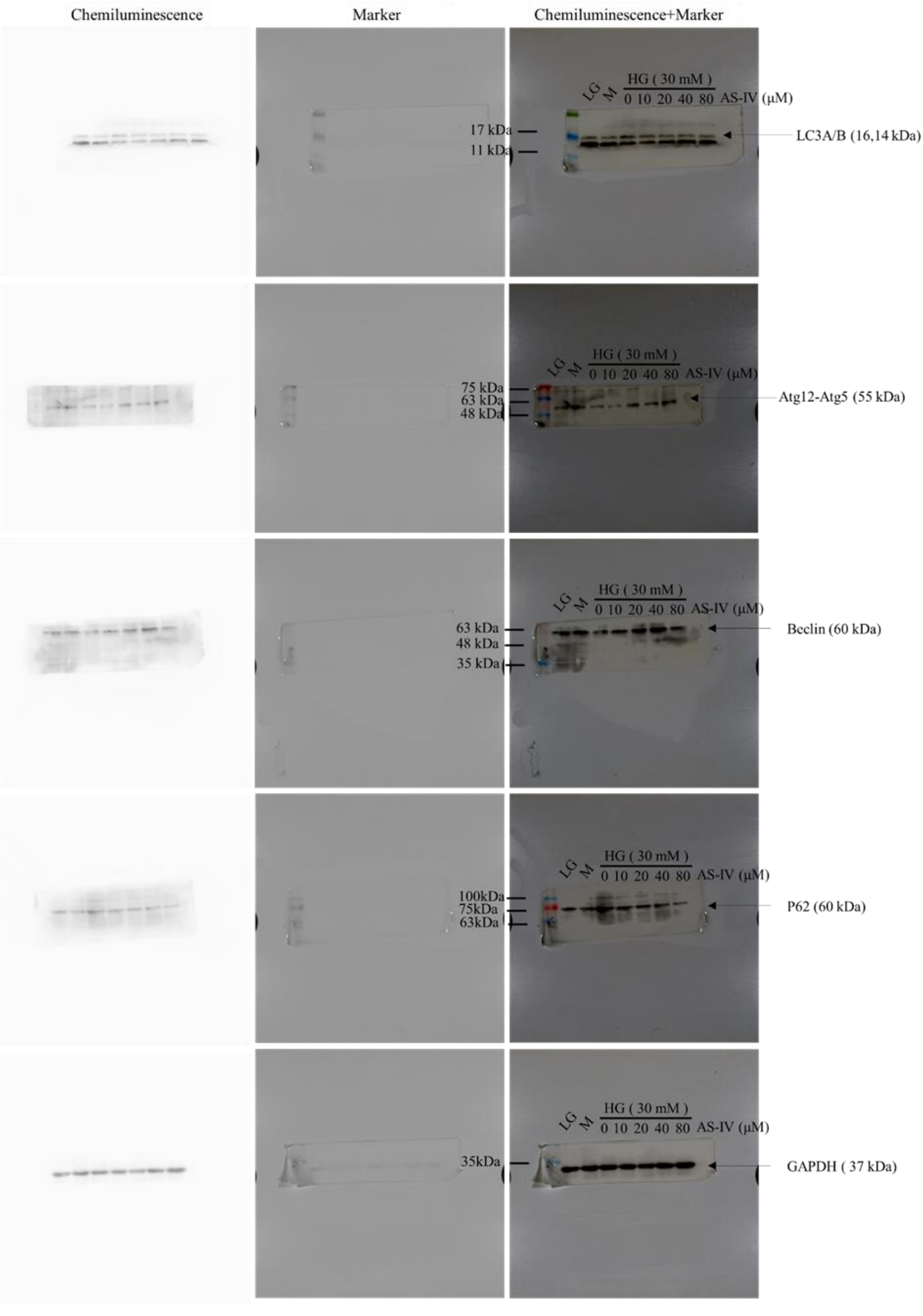

Chemiluminescence
Marker
Chemiluminescence+Marker
LG
M
HG ( 30 mM )
0 10 20 40 80 AS-IV (μM)
17 kDa
11 kDa
LC3A/B (16,14 kDa)
75 kDa
63 kDa
48 kDa
Atg12-Atg5 (55 kDa)
63 kDa
48 kDa
35 kDa
Beclin (60 kDa)
100kDa
75kDa
63kDa
P62 (60 kDa)
35kDa
GAPDH ( 37 kDa)

**D**

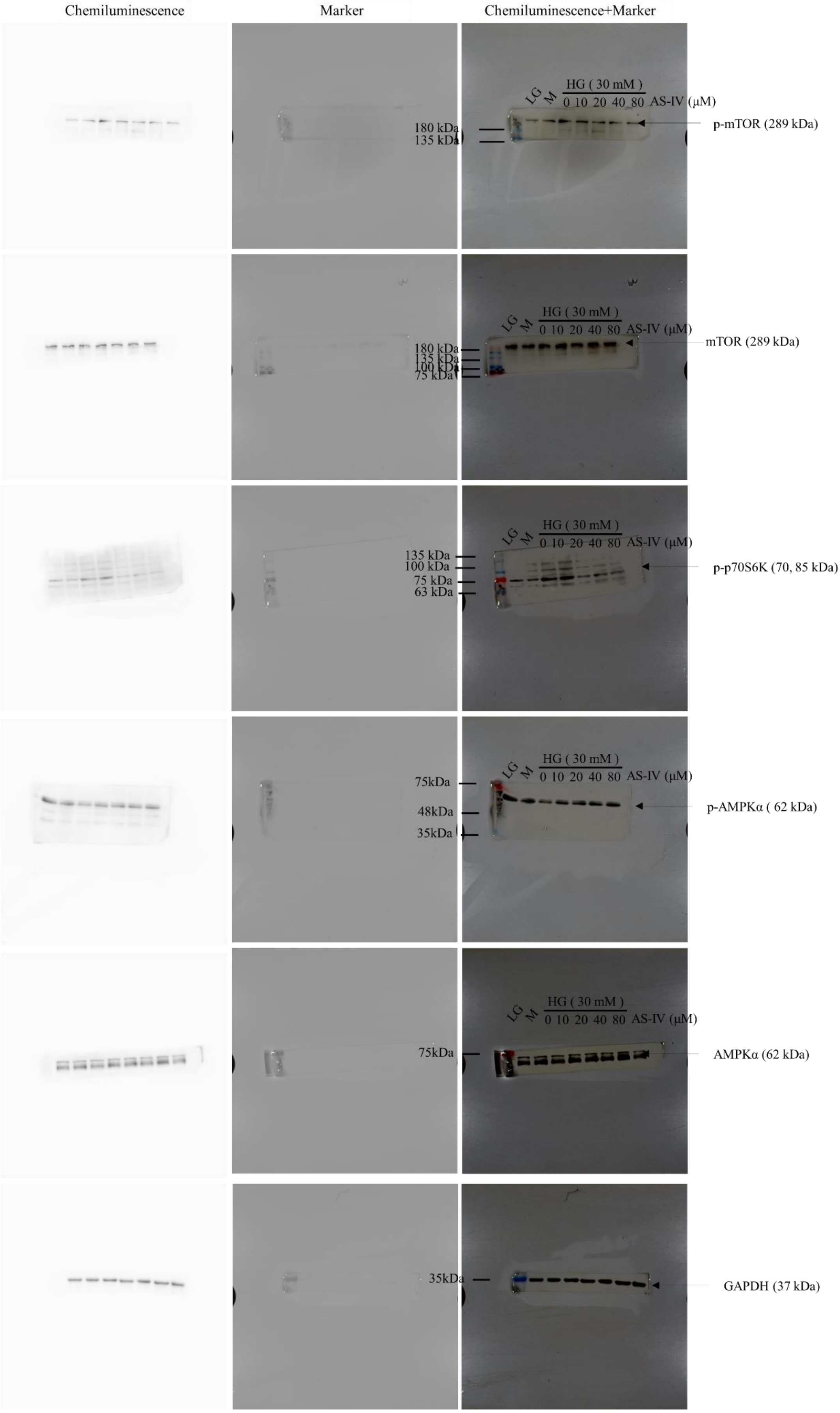
Chemiluminescence
Marker
Chemiluminescence+Marker
LG
M
HG ( 30 mM )
0 10 20 40 80 AS-IV (μM)
180 kDa
135 kDa
p-mTOR (289 kDa)
180 kDa
135 kDa
100 kDa
75 kDa
mTOR (289 kDa)
135 kDa
100 kDa
75 kDa
63 kDa
p-p70S6K (70, 85 kDa)
75kDa
48kDa
35kDa
p-AMPKα ( 62 kDa)
75kDa
AMPKα (62 kDa)
35kDa
GAPDH (37 kDa)

**Supplementary Figure S9. The original blots for cropped blots presented in Figure 8.** (A) Prestained dual color protein molecular weight marker. (B) The original blots for panel A presented in Figure 8 in the main text. (C) The original blots for panel D presented in Figure 8 in the main text. (D) The original blots for panel F presented in Figure 8 in the main text. LG, low glucose; M, mannitol; HG, high glucose.

A

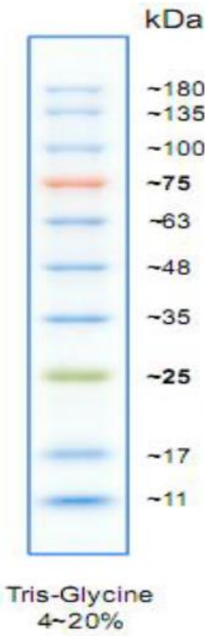
kDa
~180
~135
~100
~75
~63
~48
~35
~25
~17
~11
Tris-Glycine
4~20%

B

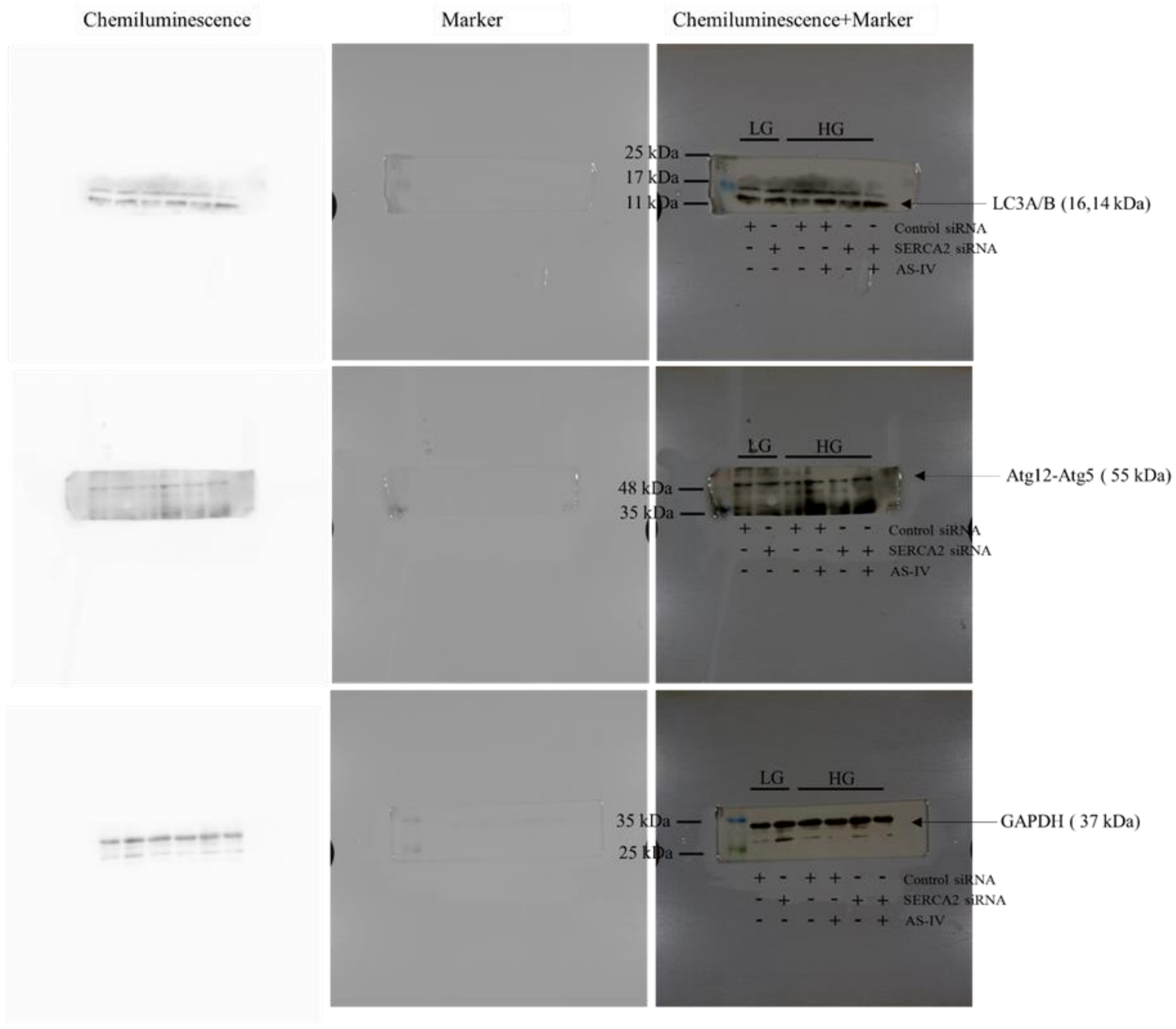
Chemiluminescence
Marker
Chemiluminescence+Marker
LG
HG
25 kDa
17 kDa
11 kDa
LC3A/B (16,14 kDa)
Control siRNA
SERCA2 siRNA
AS-IV
48 kDa
35 kDa
Atg12-Atg5 ( 55 kDa)
35 kDa
25 kDa
GAPDH ( 37 kDa)

C

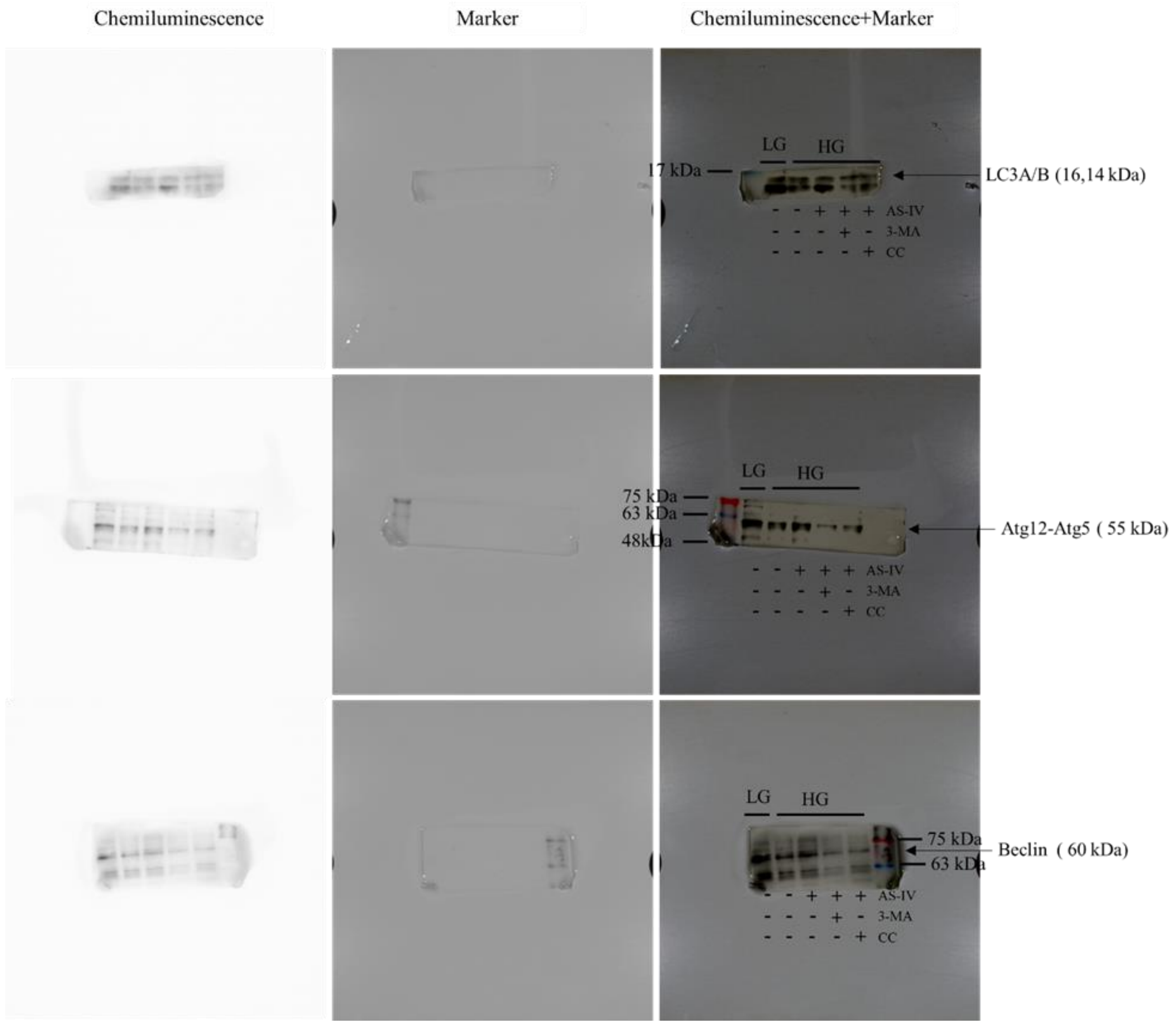
Chemiluminescence
Marker
Chemiluminescence+Marker
LG
HG
17 kDa
LC3A/B (16,14 kDa)
AS-IV
3-MA
CC
75 kDa
63 kDa
48kDa
Atg12-Atg5 ( 55 kDa)
Beclin ( 60 kDa)

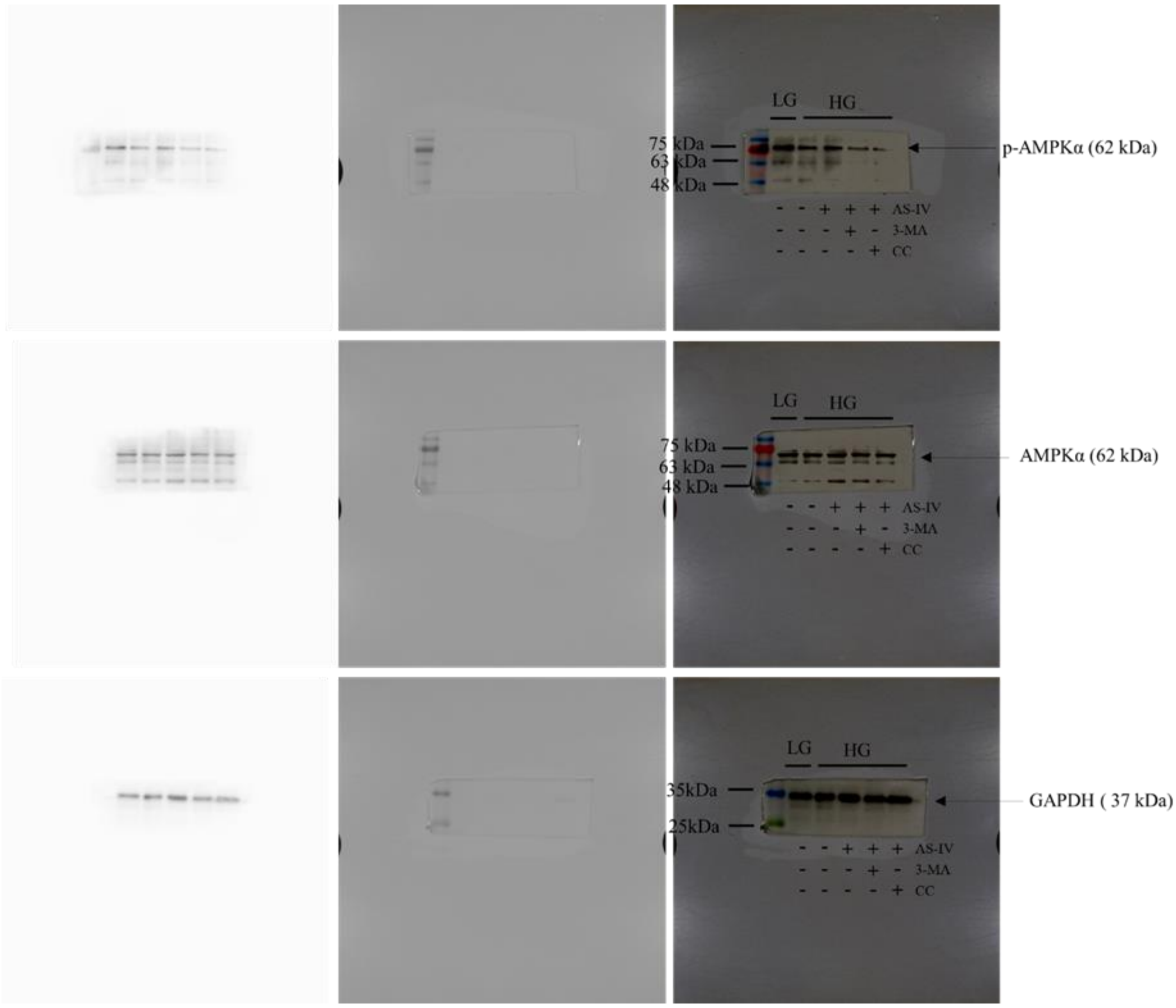


**Supplementary Figure S10. The original blots for cropped blots presented in Figure 9.** (A) Prestained dual color protein molecular weight marker. (B) The original blots for panel A presented in Figure 9 in the main text. (C) The original blots for panel C presented in Figure 9 in the main text. LG, low glucose; HG, high glucose. 3-MA, 3-methyladenine; CC, compound C.
